# The scent of attraction and the smell of success: crossmodal influences on person perception

**DOI:** 10.1186/s41235-021-00311-3

**Published:** 2021-06-26

**Authors:** Charles Spence

**Affiliations:** Crossmodal Research Laboratory, Department of Experimental Psychology, University of Oxford, Anna Watts Building, Oxford, OX2 6BW UK

**Keywords:** Scent, Fragrance, Crossmodal, Multisensory, Attraction, Person perception

## Abstract

In recent decades, there has been an explosion of research into the crossmodal influence of olfactory cues on multisensory person perception. Numerous peer-reviewed studies have documented that a variety of olfactory stimuli, from ambient malodours through to fine fragrances, and even a range of chemosensory body odours can influence everything from a perceiver’s judgments of another person’s attractiveness, age, affect, health/disease status, and even elements of their personality. The crossmodal and multisensory contributions to such effects are reviewed and the limitations/peculiarities of the research that have been published to date are highlighted. At the same time, however, it is important to note that the presence of scent (and/or the absence of malodour) can also influence people’s (i.e., a perceiver’s) self-confidence which may, in turn, affect how attractive they appear to others. Several potential cognitive mechanisms have been put forward to try and explain such crossmodal/multisensory influences, and some of the neural substrates underpinning these effects have now been characterized. At the end of this narrative review, a number of the potential (and actual) applications for, and implications of, such crossmodal/multisensory phenomena involving olfaction are outlined briefly.

## Significance statement

People have been wearing fragrance for millennia in the belief that masking their body odour will help them to look more attractive. Empirical studies of the crossmodal influence of ambient odours, personal fragrances, and chemosensory body-related odours on multisensory perception are, though, a much more recent phenomenon. A large body of research now shows that the presence of odour can indeed influence person perception through a range of mechanisms from mood-induction to crossmodal affective/semantic priming, and changes in arousal. Attractiveness judgments would appear to be influenced to a greater extent than other judgments about people by the presence of a pleasant (as compared to an unpleasant) scent. Establishing the most appropriate experimental methods by which to support claims around the efficacy of fragrance is of great commercial interest to the fragrance and home and personal care industries. At the same time, however, the methodological decisions in laboratory research designed to maximize the likelihood of observing a crossmodal effect on visual judgments of person perception often reduce the ecological-validity of the experimental designs. As such, the real-world relevance of much of the research that has demonstrated a crossmodal effect of olfactory cues (no matter whether person-related or ambient) on person perception can be questioned. This review critically evaluates the extensive literature on the olfactory modulation of person perception and highlights a number of the peculiar (or idiosyncratic) aspects of the underpinning experimental designs. At the same time, a number of specific suggestions for future research are also raised.

## Introduction

The sensory cues that happen to be presented in one modality have often been shown to influence our perception of those stimuli presented in a different sensory modality (Spence, 2021a). So, for example, the presence of pleasant versus unpleasant olfactory stimuli influence people’s ratings of everything from paintings to pictures (Wrzesniewski et al., 1999; see Spence, 2002, 2020a, for reviews). Similarly, olfactory cues have also been demonstrated to influence various aspects of person perception, such as attractiveness (Demattè et al., 2007), gender (Zhou et al., 2014), and affect. At the same time, however, it turns out that an individual’s personal odour profile provides a surprisingly rich source of chemosensory information about various aspects of their health (or disease) status (Shirasu & Touhara, 2011), their ovulatory status (in the case of fertile women; Havlíček et al., 2006; Singh & Bronstad, 2001), as well as about certain aspects of their personality (e.g., Sorokowska et al., 2012, 2016). For instance, according to Olsson et al. (2014), human body odour contains an early chemosensory cue of sickness (see also Moshkin et al., 2012). Meanwhile, different chemosensory signals have been associated with different emotions such as fear and anxiety (de Groot et al., 2012). A person’s body odour can also be influenced by aspects of their diet (Fialová et al., 2013; Havlicek & Lenochova, 2006), while single men have also been shown to have stronger body odour than partnered men, attributable to their higher levels of testosterone (Mahmut & Stevenson, 2019).

Even our choice of personal fragrance turns out not to be as random as it might first appear, and hence may also reveal more about us than one might realize (Allen et al., 2016; Janssens & De Pelsmacker, 2009; Martins et al., 2005; Milinski & Wedekind, 2001). And, one step further removed from the person themselves (and the fragrances that they choose to wear), ambient (mal)odours have sometimes been shown to influence our ratings of others too (Rotton, 1983). As such, while an individual’s olfactory signature, no matter whether natural or synthetic, presumably constitutes one component of multisensory person perception, the more general influence of synthetic ambient olfactory stimuli on person perception (as assessed visually) may better be considered to be a crossmodal phenomenon instead.

Given that olfactory cues have been shown to influence people’s visual judgments of everything from paintings to pictures and portraits (Banks et al., 2012; see Spence, 2020a, for a review), one might be tempted to wonder whether there is actually anything special about the crossmodal effects of olfaction that have been documented in terms of multisensory person perception? One difference that is immediately worth highlighting here relates to the fact that humans do have a biologically relevant natural aroma (even if they typically choose to hide it; Largey & Watson, 1972),^1^ while paintings, pictures, photos, and portraits are essentially odourless (and, what is more, are not expected to smell; though see Braun et al., 2016). That said, they are also typically silent; and yet what we hear has been shown to affect our ratings of such unimodal visual stimuli (Gerdes et al., 2014). Given the widespread and longstanding suggestion that wearing fragrance can make us look more attractive to others,^2^ one might therefore wonder why more works of art aren’t scented (e.g., at the National Portrait Gallery in London, for example). Would the Venus de Milo statue or the Mona Lisa painting be rated as any more beautiful were a matching fragrance to be been released in The Louvre galleries in Paris where the works are displayed? Note that this suggestion is not as far-fetched as perhaps it might seem, given that, in 2019, The Louvre commissioned a number of top perfumers to create fragrances for eight of the works in the collection (Bremner, 2019; Spence, 2020a).^3^

One area of particular interest concerns whether the multisensory/crossmodal influence of olfactory stimuli on face/person perception is specific, or whether instead much the same effects can potentially be documented by the presence of any other atmospheric cue, for example, the emotional affect, or arousal that is often elicited by listening to music, or the attractiveness of the environment in which the faces happen to be rated (Maslow & Mintz, 1956). Here, for example, there is research showing the crossmodal impact of emotional music on the perception of, and memory for, faces (Proverbio et al., 2015). Indeed, the arousal that can be induced by listening to music has been shown to influence our ratings of the attractiveness of faces (e.g., Marin et al., 2017; May & Hamilton, 1980; cf. Risso et al., 2021, for the suggestion that olfactorily induced changes in arousal may also be one of the mechanisms by which scent may influence judgments of visual attractiveness). Given that ambient olfactory stimuli influence our mood (Herz, 2002; Schiffman, 1974; Spence, 2020c; Vernet-Maury et al., 1999), such emotional crossmodal influences on stimulus processing are likely to be relatively nonspecific (Pourtois et al., 2013), meaning that they may affect our evaluation of many different kinds of perceptual stimuli (i.e., not just faces). At the same time, however, it is important to note that the presence of scent not only influences a perceiver’s impression of other people, it may also affect their impression of themselves, possibly enhancing their self-confidence (and, as we will see later, this may also be picked up by others too). At the same time, however, it is also important to differentiate the concept of crossmodal olfactory–visual interactions with olfactory influences on person perception, which although partly visual are also importantly social, emotional, and cultural (cf. Cerulo, 2018; Moeran, 2007).

Evaluating the evidence on fragrance effects on person perception, and the underlying cognitive mechanisms, where they are known, or have been suggested, may also help those wishing to critically evaluate the popular psychology literature that has developed around the suggestion that perfume can be used as an effective tactic of impression management in social and organizational settings (Baron, 1988; Levine & McBurney, 1986; Lobmaier et al., 2020; Newsweek, 1984; Zemke & Shoemaker, 2007), in non-verbal communication, and in order to engage in behavioural, or sensory, nudging (Baron, 1980; Cowley et al., 1977; De Lange et al., 2012; Ebster & Kirk-Smith, 2005; Gueguen, 2001; Gustavson et al., 1987; Hold & Schleidt, 1977; Hirsch, 1993; Hirsch & Gruss, 1999; Kirk-Smith & Booth, 1980; Liljenquist et al., 2010; Razran, 1940; Sczesny & Stahlberg, 2002; Taylor, 1968, p. 53).^4^ As might have been expected, and as we will see later, there has also been extensive commercial interest in supporting claims around the role of fragrance in attraction/attractiveness—i.e., both in terms of a fragrance’s ability to boost the wearer’s self-confidence, but also to influence how they are perceived by others (Berliner, 1994; Hirsch, 2006). But, one might ask, are all the attributes/dimensions of person perception equally affected by the presence of scent/malodour, or are some judgments more malleable/important than others? And, if that is the case, how should any such differences be accounted for?

Furthermore, while significant crossmodal effects of olfactory stimuli on visual ratings of the attractiveness of those people shown in photographs have been demonstrated in many studies, not all studies have demonstrated such crossmodal effects (see Cann & Ross, 1989; Novak et al., 2015, for a couple of null results). In the current academic climate, this naturally leads to concerns about power and reproducibility (Iso-Ahola, 2017; Open Science Collaboration, 2015; see also Syrjänen et al., 2021). As such, one of the other important questions to be addressed by this review is to try and identify some of the key factors that may be responsible for determining whether or not a crossmodal influence of olfaction on vision will be observed. Potentially relevant here, and as we will see time and again throughout his review, it is often unclear what exactly the link between the fragrance and the people that the participants were being asked to judge actually was. One might have imagined that establishing some meaningful connection between scent and sight would be a prerequisite for the former to influence the latter. However, somewhat surprisingly, that turns out not to be the case.^5^

### Outline

In the sections that follow, I will first review the literature that has investigated the influence of odour on person perception (“The influence of odour on person perception” section). First, the evidence concerning olfactory influences on attractiveness but also on a number of other qualities (such as beauty, charm, familiarity, intelligent, socially competent, and confidence) is reviewed. The various explanations for these crossmodal effects of olfaction on vision are summarized and the possible influence of visual stimuli on olfactory stimuli is also briefly discussed. In the “Limitations/peculiarities of crossmodal research on olfaction on person perception” section, I highlight a number of salient limitations/peculiarities in the literature examining crossmodal influences of olfaction on person perception. This includes everything from the familiarity (or otherwise) of the people whose pictures are being judged, through to the timing of the stimuli, and the lack of any explanation (to the participants) of what the connection between the scents they are smelling and the faces they are judging is. In “The influence of a person’s natural body odour on multisensory person perception” section, I move on to consider the influence of a person’s natural body odour on multisensory person perception, before summarizing the limited evidence regarding the impact of a person’s odour/fragrance, on their perception of themselves (see the “Olfactory influence on perception of the self” section). The “Attractiveness as a multisensory construct” section  briefly discusses the notion of multisensory attractiveness, and how the different sensory cues to person perception may be combined. “Commercial interest in claims around the effects of fragrance on attractiveness” section  switches briefly to consider the commercial opportunities around supporting claims concerning fragrance’s effect on person perception. Finally, in the “Conclusions” section, conclusions are summarized and directions for future research, as well as potential applications and implications of this research, are discussed.

## The influence of odour on person perception

At the outset of this narrative review, it is important to note that there are multiple different kinds of situation in which olfactory stimuli may be present while we engage in person perception. On the one hand, one might be interested in the question of what role ambient (mal)odour plays in terms of personal attraction. However, there has been an explosion of research looking at the question of what a person’s body odour, or the use of personal fragrance (when presented prior to and/or while faces are being judged), may do in terms of the multisensory impression that they create, as well as how they make people feel about themselves. The evidence relevant to each of these cases below is reviewed in the following sections.

### The influence of ambient scent on person perception

In those studies where an ambient scent has been introduced into a testing room, the natural presumption would be that whoever experiences the smell would attach it to the location, rather than necessarily to the individual faces that are flashed up briefly on the screen (as is typically the case in laboratory studies in this area). Nevertheless, despite the lack of a unity judgment (Chen & Spence, 2017), the (un)pleasantness of the ambient odour has nevertheless still been shown to influence judgments of interpersonal attraction, based on other people’s attitudinal questionnaire responses (Rotton et al., 1978; cf. Sczesny & Stahlberg, 2002; Experiment 1) or else, more commonly, their photos (e.g., Cann & Ross, 1989; Rotton, 1983). For example, Rotton investigated the influence of ambient malodour on 48 students’ ratings of four photographic negatives (taken from the school yearbook), four paintings, and four persons described by adjectives. Ethyl mercaptan (C_2_H_5_SH) served as the ambient malodour. This volatile chemical is described as smelling of rotting cabbage or sewer gas, though when presented in its pure form, as in Rotton’s study, it is apparently even more unpleasant/revolting. Hence, a highly aversive olfactory stimulus was used, with no meaningful connection to any of the visual stimuli that the participants had to rate. An elaborate ruse designed to make the presence of the malodour seem accidental was performed at the start of the experimental session. A between-participants experimental design was used with the participants rating the 12 visual stimuli once in either an odourless or else in the very malodorous room. The photos were rated on seven-point scales anchored with the words ‘zestful’ and ‘weary’ or ‘content’ and ‘irritable’. The people shown in the photographs were rated numerically (by about 5%), but not significantly (*p* < 0.07) as having less ‘energy’ and significantly lower ‘well-being’ (c. 10% change; *p* < 0.01) in the polluted than the unpolluted room (see Table 1 for a chronological summary of psychophysical research investigating the crossmodal influence of olfactory stimuli on ratings of the attractiveness and other personal attributes of those shown visually). These early results therefore support the claim that ambient malodour affects person perception. Note also the small sample sizes involved in a number of the studies.

**Table 1:** Chronological summary of research investigating the crossmodal influence of olfactory stimuli on people's ratings of other people's faces (typically unknown others shown in photos)

Study	Participants	Olfactory stimuli	Presentation of odorants	Face rating	Comments
Rotton (1983)	24 males and 24 females	Ethyl mercoptan (very −ve) or NO	Between- sessions	Energy and well-being of 2 male and 2 female faces	Ambient malodour only reduced ratings of well-being significantly
Cann and Ross (1989)	63 males	*Island Gardenia* by *Jovan* (+ve)/Ammonium sulfide (−ve)/NO	Between- participants	Attractiveness of 50 female faces	No effect of pleasantness of ambient odour on ratings
Kirk-Smith and Booth (1990)	?	*Shalimar* perfume(+ve)/Banana essence (+ve)/NO	Between- participants?	Sexiness of 1/2 naked torsos and softness	*Shalimar* perfume led to sig. change in sexiness ratings. Banana odour = no effect
Bensafi et al. (2002)	14 females	Floral fragrance (+ve)/NO	Blocked?	32 female faces rated pleasant or not	Discrete either/or choice and absence of unpleasant odour may help to explain null results
Hirsch (2006)	37 (males and females)	Pink grapefruit/Grape/Cucumber/NO (Pink grapefruit + vanilla + baby powder)	Between- participants	Age of 20 male and female faces	Only pink grapefruit lowered age, especially for men rating women
Demattè et al. (2007)	16 females	Geranium and *Lynx* fragrance (+ve)/Body odour and Rubber (−ve)/NO	Trial-by-trial	Attractiveness of 40 male faces	Unpleasant odours lowered attractiveness ratings. No effect of body relevance
Li et al. (2007)	30 (males and females)	Lemon (+ve)/ ethereal (neutral)/sweat (−ve)	Trial-by-trial	Likability of 80 faces (sex not specified)	Assimilation in non-conscious group, no effect of odour in conscious group
Capparuccini et al. (2010)	50 males and 50 females	*Pi neo* (M) (+ve)/*Angel or Demon* (F) (+ve)	Between- sessions	10 characteristic of 5 M and 5 F faces (neutral emotion):	Beauty ratings enhanced most by gender congruent (with respect to participant) compared to gender-incongruent scents. No effect for gender neutral ratings (familiarity and confidence)
McGlone et al. (2013)	16 females	*Lynx* fragrance (+ve)/Synthetic body odour (−ve)/NO	Trial-by-trial	Attractiveness of 20 male faces	Unpleasant odours sig. lowered ratings relative to NO and +ve fragrance condition
Marinova and Moss (2014)	36 females	*In Motion* perfume by *Hugo* Boss (M) (+ve)/*Ghost* perfume by *Ghost* (F) (+ve)/NO	Between- participants	Attractive, reliable, outgoing, intelligent, wealthy, and socially competent of 15 male faces	Gender-incongruent female perfume lowered attractiveness ratings only for medium attractive men compared to congruent scent
Seubert et al. (2014)	6 males and 12 females	5 odours from 100% fish (−ve) to 100% rose (+ve)	Trial-by-trial	Attractiveness and age of 8 female neutral faces morphed to show more versus less wrinkles and blemishes than comparison	Odour valence affected attractiveness but −ve odour may have distracted in age task
Cook et al. (2015)	20 (males and females)	Methylmercaptan (−ve)/Jasmine (+ve)/NO	Trial-by-trial	Pleasantness of 18 male and 18 female neutral faces	Pleasant aroma led to higher pleasantness ratings than NO, which, in turn, was higher than for unpleasant rotton cabbage odour
Novak et al. (2015)	8 males and 8 females	NO/Lilac (+ve) /Sweat (−ve)	Blocked	Emotion of 4 female and 4 male dynamic face stimuli	Null results in this pre-registered study
Cook et al. (2017)	20–23 (males and females)	Methylmercaptan (−ve)/Jasmine (+ve)/NO	Trial-by-trial	Pleasantness of 30 happy and 30 disgusted male and female faces	The −ve odour significantly lowered pleasantness ratings relative to +ve odour for happy and disgusted faces
Cook et al. (2018)	26–28 (males and females)	Methylmercaptan (−ve)/Jasmine (+ve)/NO	Trial-by-trial	Pleasantness of 45 male and 45 female neutral faces	Simultaneous presentation of face and −ve odour led to near sig. > reduction in pleasantness of faces than sequential presentation
Risso et al. (2021)	6 males and 6 females	NO/Liquorice (M) (+ve)/Caramel (F) (+ve)	Trial-by-trial	Attractiveness of 30 male and 30 female faces	Gender-incongruent food odour lowered ratings more than gender-congruent food odour

M, male; F, female; + ve/neutral/ − ve refers to valence of odour; ?, uncertain; NO, no odour condition

By contrast, however, Cann and Ross (1989) failed to demonstrate any such crossmodal effect of malodour on facial attractiveness. In particular, the male college students in the latter study had to rate a series of pictures of female faces while in the presence of either a pleasant fragrance (*Island Gardenia* by *Jovan* which had a pleasant flowery aroma) or an unpleasant ambient odour (ammonium sulphide), or else in the absence of any olfactory stimulus. The photographs were rated using a 10-point attractiveness scale (anchored with the labels ‘extremely unattractive’ and ‘extremely attractive’) in a room that had been scented prior to the participants’ arrival. In this case, the presence versus absence of an ambient odour had absolutely no effect on the participants’ judgments of facial attractiveness. It should, however, be noted that the null effects reported in this case might well be attributable to the use of ambient room fragrancing, thus potentially reducing the likelihood that the participants would necessarily have associated the unchanging room odour with the sequentially presented faces. That is, there was little reason for the participants to believe that the faces and odours were connected, or that they belonged together and so should be unified (see Chen & Spence, 2017, for the importance of the unity effect in multisensory perception). Indeed, Cann and Ross (1989, p. 96) explicitly state that as the groups of four participants were being escorted to the testing room: ‘the experimenter apologized for the odor and claimed to have no knowledge about what had gone on before in the room to create the smell. This was intended to minimize curiosity about the odors’.

The rapid habituation of the olfactory system, especially under conditions of high visual load (see Forster & Spence, 2018) means that it is difficult to rule out the possibility that the odours might simply not have been consciously perceived for much of the latter part of the experimental session in Cann and Ross’s (1989) study.^6^ The between-participants nature of the experimental design may also have contributed to the null results. Nevertheless, Cann and Ross (1989, p. 99) ended up concluding that: ‘it may be that olfactory stimuli produce no reliable effects on judgments of attractiveness’. At the same time, however, they also allow for the fact that if olfaction’s effect on attractiveness ratings were to be based on mood enhancement then their study failed to demonstrate a robust effect on mood (despite the fact that ammonium sulphide is typically described as a very unpleasant olfactory stimulus).

Kirk-Smith and Booth (1990) conducted a study (reported in a book chapter) in which impregnating a face-mask with *Shalimar* perfume,^7^ resulted in both male and female observers rating half-torso clothed photographs of males and females as looking significantly sexier and softer as compared with a no-perfume condition. By contrast, those participants who wore a face-mask that had been impregnated with banana essence exhibited no such crossmodal effect, perhaps due to the incongruence between the odour that they were exposed to and the photos that they had been requested to rate.^8^ However, it is worth noting that the prolonged presentation of the odours meant that their presence may have elicited a change in participants’ mood. Indeed, the participants even rated themselves as feeling sexier after exposure to the *Shalimar*-impregnated face mask. Consequently, it is difficult to separate out the direct crossmodal effects of the presence of the odour on judgments of the people seen in the photographs from the more indirect effects that extended exposure to the perfume may have had on the mood of the participants, which, in turn, could have given rise to the behavioural effects that were reported. This possible explanation for the findings was one that Kirk-Smith and Booth themselves acknowledged.

Bensafi et al. (2002) conducted an event-related potential (ERP) study in which female volunteers had to discriminate whether a sequence of female faces were pleasant or unpleasant. Ratings were either carried out following the presentation of a pleasant floral odour for 5 s or else in a no odour baseline condition. (Note that no fragrance was presented while the face was being displayed on the screen.) Although the methods are a little unclear, it would appear as though the presence versus absence of the floral odour was switched half way through each block of 32 trials. However, there was virtually no difference in the percentage of faces rated as pleasant in the pleasant floral odour condition (*M* = 36.8%) as compared to the no odour baseline condition (*M* = 36.2%; comparison, n.s.). In this case, though, the use of a dichotomous response (rather than a more fine-grained rating of facial attractiveness as has been used in the majority of other studies) may have been at least partially responsible for the null result. At the same time, and as we will also see later, unpleasant odours generally tend to have a larger effect on attractiveness ratings than pleasant fragrances when compared to a neutral baseline (e.g., see Cook et al., 2018; Demattè et al., 2007; Li et al., 2007). In their study, Bensafi et al. also explicitly state that they made no mention to their participants that any scent would be presented, thus presumably leaving their participants uncertain of the crossmodal connection, if any, between the sensory stimuli that they experienced.

Generally speaking, the assumption in the literature would appear to have been that mood-based effects of olfactory stimuli on person perception are more likely to be observed in those situations in which the participants are exposed to a particular fragrance for a prolonged period of time (e.g., over a block of trials, or over an entire experimental session in between-participants studies). At the same time, however, it is worth remembering that any olfactory adaptation effects might well be expected to eliminate the effect of olfaction on mood over time. By contrast, in those studies that will be described in the next section, where the fragrance/smell has more often changed randomly on a trial-by-trial basis, there would seem to be little reason to believe that mood-induction provides a likely explanation for any crossmodal effects of odour that have been documented (as the timecourse is simply not appropriate).

### The influence of hedonically valenced smells on multisensory person perception

**Fig. 1:**
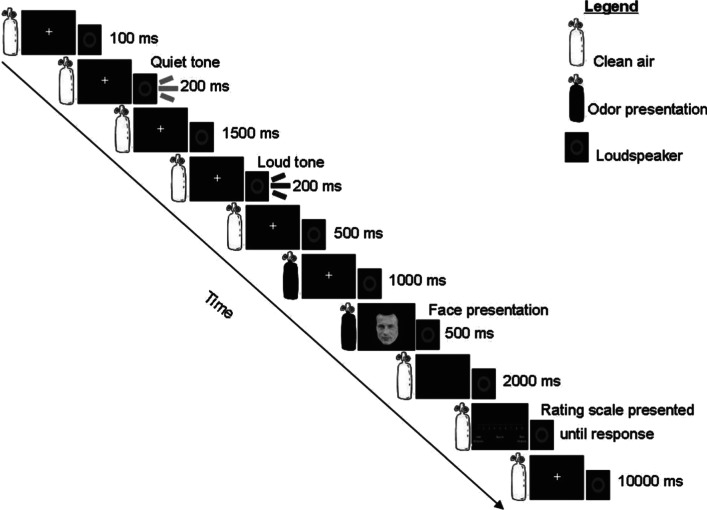
Timeline describing the experimental procedure used in Demattè et al.’s (2007) experiment to demonstrate the crossmodal influence of pleasant versus unpleasant olfactory stimuli on young women’s ratings of men’s faces. Note that the olfactory and visual stimuli were varied randomly on a trial-by-trial basis. Notice also how, like in the majority of other studies of olfactory influences on visual attractiveness, the onset of the olfactory stimulus occurred prior to that of the visual stimulus. [Reprinted with permission from Demattè et al. (2007, Figure 1)]

In what was perhaps the first trial-by-trial study of the crossmodal influence of fragrance on ratings of visual attractiveness to have been published, Demattè et al. (2007) had 16 young females participants (mean age of 26 years) rate a series of 40 cropped youthful male faces on a 9-point visual scale (anchored with ‘most attractive’ and ‘least attractive’). On each trial, a fragrance (either pleasant or unpleasant, body-related or not; specifically *Lynx* deodorant,^9^ geranium, body odour—BO, and rubber)^10^ or else no fragrance was presented prior to a randomly selected face, which the female participants had to rate (see Fig. 1). On each and every trial, the participants reported whether or not a fragrance was present prior to the onset of the visual stimulus, thus ensuring that the participants had paid attention to the olfactory stimuli. Ratings of the male faces were significantly influenced by the pleasantness of the fragrance (by c. 5%), though (contrary to the experimenters’ expectations) the body-relevance factor had no influence on ratings. Rather, it turned out that presenting either of the two unpleasant odour(s) led to a significant reduction in the attractiveness of the male faces when compared to the neutral/no odour, or pleasant odour conditions (with participants’ ratings for the latter conditions not differing significantly). Given that the onset of the olfactory stimulus occurred 500 ms prior to the onset of the visual stimulus (with both stimuli then being presented together for a further 1000 ms), these results should perhaps best be considered in terms of crossmodal affective priming (about which, more below).

**Fig. 2:**
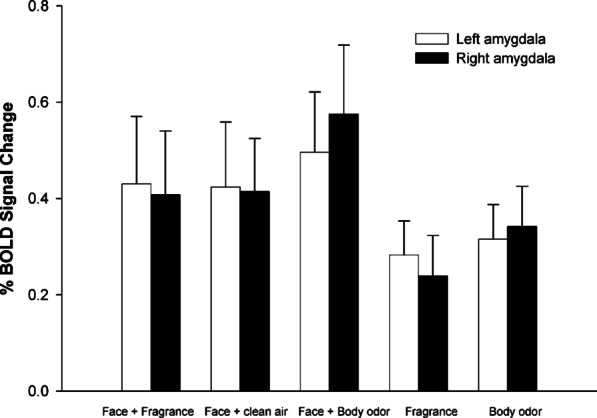
Summary of neuroimaging results from McGlone et al.’s (2013) follow-up to Demattè et al.’s (2007) psychophysical study. The graph showing the average (*n* = 16 participants) percentage BOLD signal change in the peak voxels in both medial and lateral OFC for each of the five experimental conditions. Error bars depict the standard error. [Reprinted with permission from McGlone et al. (2013, Figure 6).]

McGlone et al. (2013) conducted a functional MRI follow-up to Demattè et al.’s (2007) psychophysical study using much the same experimental procedure but this time with a new group of young female participants lying in the brain scanner rather than seated comfortably in the psychophysics laboratory. The same basic crossmodal (affective priming) effect of odour pleasantness on ratings of facial attractiveness was observed (in this case only the Lynx and artificial BO were used). The faces were rated as roughly 8% more attractive when preceded and accompanied by the smell of Lynx as compared to when accompanied by the synthetic body odour. Once again, there was no significant difference between the pleasant fragrance and the no fragrance control condition. A shift in the focus of activation within the neural representation of attractiveness that has been documented in orbitofrontal cortex (OFC), was observed. Note that the OFC has been implicated in encoding the reward value of stimuli (e.g., Kahnt et al., 2010). More specifically, when the faces were paired with the pleasant fragrance, increased activation was observed in the medial part of orbitofrontal cortex and ventral striatum. By contrast, when the same faces were paired with the unpleasant odour instead, the activation seen in the insula and amygdala, which is known to be involved in the processing of aversive stimuli, increased (see Fig. 2). These results were taken to support a genuine crossmodal perceptual effect of olfactory cues on the visual perception of attractiveness (i.e., rather than some kind of olfactorily induced response bias or halo effect).

**Fig. 3:**
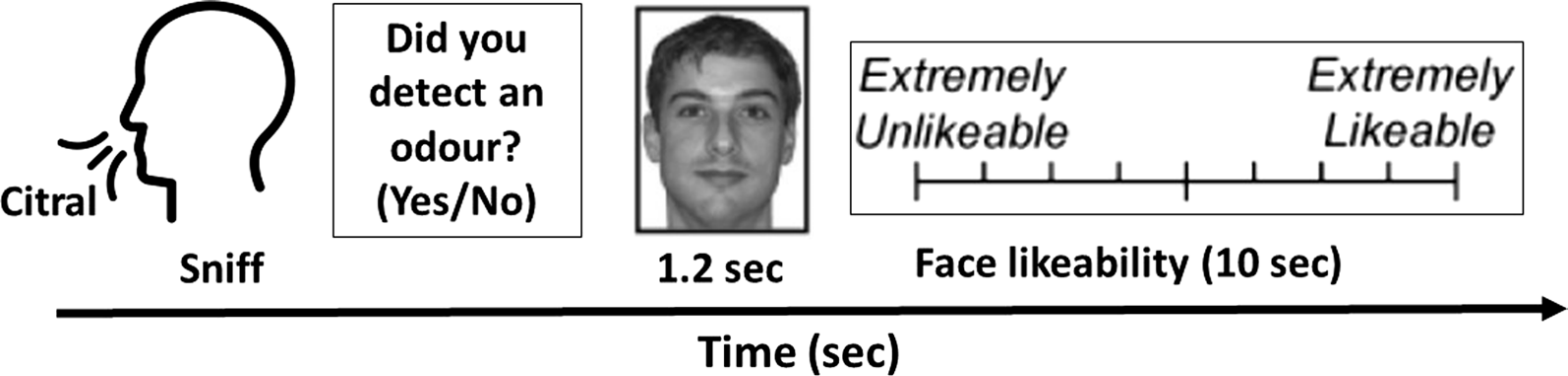
Schematic illustration of the experimental paradigm used by Li et al. (2007). Initially, each participant’s odour-detection thresholds were established using an ascending-staircase procedure (not shown). Next, the participants had to sniff a bottle, indicate whether or not it contained an odour, view a face stimulus, and thereafter rate the face in terms of its likeability. [Redrawn from Li et al. (2007, Figure 1).]

Li et al. (2007) demonstrated that ratings of the likability of a selection of faces displayed on a computer monitor were influenced by the presentation of a scent, especially if the latter was delivered at a subliminal level. Participants rated the likeability of neutral faces (using a visual analog scale—VAS—anchored with ‘extremely unlikeable’ and ‘extremely likeable’) after smelling near-threshold pleasant (‘citral’), neutral (anisole), and unpleasant odorants (valeric acid). Note that when presented at suprathreshold levels, these stimuli are often described as smelling of lemon, ethereal, and sweat-like, respectively. In the experiment itself, the participants first sniffed one of four bottles and reported whether or not they had detected an odour. Immediately thereafter, the participants rated the likeability of one of 80 emotionally neutral faces presented on a computer monitor for 1200 ms (see Fig. 3). The valence of the odour significantly shifted likeability ratings only for those participants who lacked conscious awareness of the olfactory stimuli, as verified by chance-level trial-by-trial performance on the odour-detection task (i.e., the 15 participants with an unadjusted *d′* prime of 0). The other group of 15 participants with an unadjusted *d′* prime of close to 1, were classed as the conscious group. In other words, across participants, the magnitude of this crossmodal priming effect decreased as the sensitivity for odour detection increased, meaning that subliminal odours had a larger impact than did the supra-threshold odours.

The sequential presentation of olfactory then visual stimuli from different locations/sources (that were semantically unrelated with the possible exception of the sweat smell) would presumably have given the participants in Li et al.’s (2007) study little reason to want to combine (or multisensorially integrate) the scent and suprathreshold face stimuli consciously. That said, it is interesting to note that in the unconscious group, only the unpleasant (i.e., possibly semantically related) sweat stimulus differed significantly from the pleasant and neutral scent conditions. However, with the limited range of olfactory stimuli used by Li et al., it is simply not possible to determine in hindsight whether this was driven by the body-relevance of the odour, by its being unpleasant, or by some other, as yet unidentified factor.

Once again, therefore, Li et al.’s (2007) results would appear to be more consistent with a crossmodal affective priming account driven primarily by the presence of the unpleasant (and in this case unnoticed) scent prime. Affective priming refers to those situations in which an affective stimulus (i.e., prime) evokes an emotional response in a perceiver/participant that is then carried over to the processing of a subsequent stimulus (i.e., target), modifying affective evaluation of the latter stimulus (Hermans et al., 1998; Murphy & Zajonc, 1993; see also Forgas et al., 1984). The 18 participants in a methodologically complex study by Seubert et al.’s (2014) presented two faces sequentially on each trial. The first face was always the standard (showing someone of middle-age), while the second has been morphed to show either 25 or 50% more or less wrinkles and blemishes. The participants initially made a speeded two-alternative-forced—choice (2AFC) judgment concerning whether the second face looked older or younger than the first. On a third of the trials, the participants had to rate how attractive the second face was (on a 100-point VAS anchored with ‘extremely unattractive’ and ‘extremely attractive’). On another third of the trials, they rated how old the face/person was (in this case, the scale was anchored with < 25 years at one end and > 60 years at the other, with 5-year tick marks added along the scale). In the remaining third of trials, the participants rated the valence of the odour that had been presented in that trial.

**Fig. 4:**
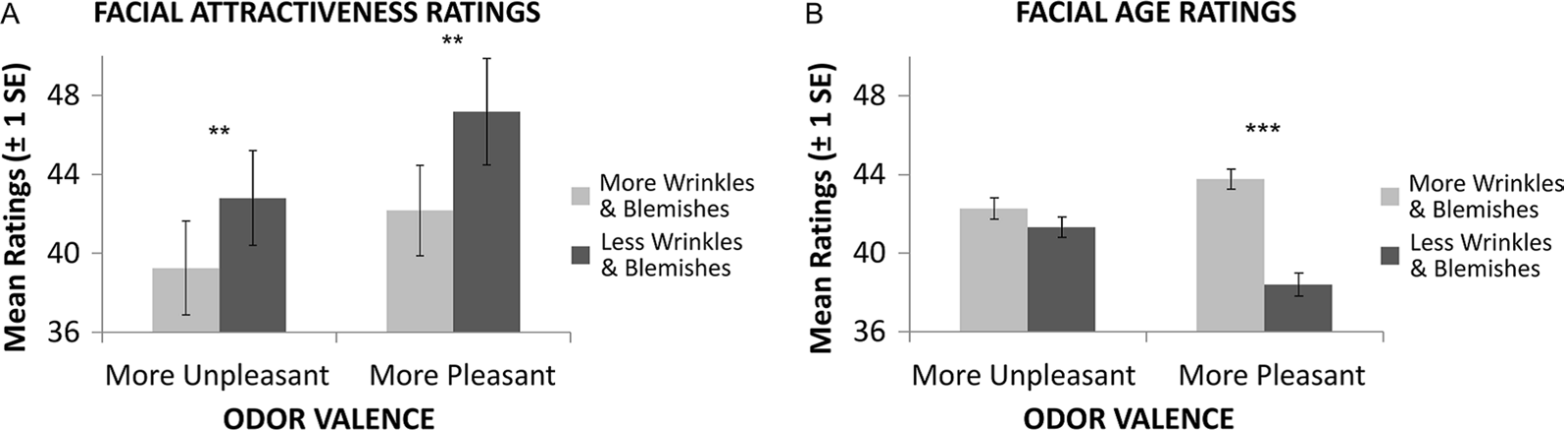
Results of factorial analyses for categorical effects of odours and facial morphing on attractiveness (**A**) and age (**B**) and ratings in Seubert et al.’s (2014) study. Ratings were provided on a visual analog scale consisting of 100 sub segments, which in the case of age was anchored at 25 years and 60 years for ecological validity. Error Bars indicate ± 1 SE, asterisks indicate significant differences as revealed by post hoc *t* tests (* = *p* 0.05, ** = *p* 0.01, *** = *p* 0.001). [Figure reprinted with permission from Seubert et al. (2014, Figure 3).]

The onset of the second face was preceded and overlapped with the presentation of one of five fragrances varying parametrically between 100% fish odour (negatively valenced) and 100% rose scent (positively valenced). As predicted, judgments of attractiveness (which rely on affective processing) were linearly affected by the valence of the concurrently presented odour, consistent with visual and olfactory cues to attractiveness being represented within a common affective neural system. By contrast, the presentation of the unpleasant odorants in the more putatively ‘cognitive-analytic’ age judgment task appeared to interfere with performance (see Fig. 4 for results). One obvious limitation with this study is that the speeded task that participants performed first on every trial presumably led the participants to weight the two attributes (age and beauty) rather differently due to the specific task demands. The extent to which this particular aspect of the experimental design may have skewed the pattern of results that were obtained is unclear. Once again, as we have seen for pretty much all of the studies reported in this section, the researchers concerned make no mention of their participants being told anything about the link between the odorants that they had been presented with and the visual stimuli that they were expected to evaluate.

A similar (affective priming) explanation would also seem to apply in the case of the influence of pleasant and unpleasant odours (jasmine vs. methylmercaptan, the latter delivering a rotten-cabbage-like odour), or a no odour baseline, on the hedonic evaluations of people’s faces that was demonstrated in a combined behavioural and ERP study reported by Cook et al. (2015). In this study, the odorant was presented for three seconds, and the picture was then presented one second after its offset. The participants rated each neutral expression face on a 101-point visual scale anchored with ‘very unpleasant’ and ‘very pleasant’. After having rated the face on each trial, the participants were then prompted to rate the intensity of the olfactory stimulus that had just been presented. The results revealed that even though the hedonic olfactory stimulus was presented before the to-be-rated face, it nevertheless still led to hedonic assimilation, with the presentation of the pleasant jasmine odour leading to the subsequently presented face being rated as significantly more pleasant (*M* = 55) than the same faces when rated in the absence of any olfactory stimulus (*M* = 53). The lowest pleasantness ratings were documented in those trials where the faces were preceded by the smell of rotten cabbage (*M* = 50; i.e., the pleasant–unpleasant difference once again amounted to a c. 5% change in ratings, just as in Demattè et al.,’s, 2007, study). In a later study, Cook et al. (2017) went on to demonstrate that ratings of the pleasantness of happy and disgusted (i.e., emotionally expressive rather than neutral) faces were also modulated by the presentation of the pleasant versus unpleasant odour that onset 1–2 s prior to and overlapped with the presentation of the face stimulus for 300 ms.

The ERP results from Cook et al.’s (2015) study revealed that the odour-induced shifts in face evaluation were associated with amplitude changes in the late (> 600 ms) and ultra-late (> 900 ms) latency epochs. The authors write that: ‘The observed amplitude changes during the ultra-late epoch are consistent with a left/right hemisphere bias towards pleasant/unpleasant odor effects’. (Cook et al., 2015, p. 1). They go on to conclude that their: ‘Results suggest that effects of pleasant odors on face evaluation were specific to the late component. During the ultra-late component, effects of pleasant and unpleasant odors were distinguished in the left and right hemispheres, respectively’. (Cook et al., 2015, p. 9). In a subsequent study, the same group of researchers reported that the negative hedonic evaluation and ERPs elicited by the unpleasant odours were both strengthened on those trials in which the olfactory and visual stimuli were presented simultaneously as compared to when the picture was presented a second after the offset of the olfactory stimulus (Cook et al., 2018). On the basis of the combined psychophysical and ERP data from the latter study, these researchers concluded that the late-positive potential (LPP): ‘may represent the strength of the effects of unpleasant odour context on face evaluation that occur as a result of the temporal association between odour and face’. (Cook et al., 2018, p. 26). Interestingly, however, and in contrast to their 2015 study using essentially the same methods, there was no longer any difference between the positive odour and neutral clean air conditions in either the sequential or the simultaneous condition (though the positive vs. negative odour comparison once again revealed a difference of c. 5% in face ratings).

### Semantic olfactory priming based on gender-congruency

All of the studies reported in the previous section used hedonically valenced odours (either positive or negative) to affectively prime participants in a crossmodal manner prior to the presentation of the face stimuli. Other researchers, meanwhile, have studied the crossmodal semantic priming of vision by olfaction by, for example, presenting the scent of an apple prior to the picture of the fruit (semantically congruent) or a car, say (the latter being semantically incongruent; see Gottfried & Dolan, 2003; Grigor, 1995; see also Grigor et al., 1999). Neuroscientists have highlighted the role played by the hippocampus and orbitofrontal cortex in establishing crossmodal connections between semantically related visual and olfactory stimuli (Gottfried & Dolan, 2003). Similarly, other researchers have shown that olfactory cues can also prime visual self-recognition (Platek et al., 2004). In the latter study, for example, the participants detected pictures of their own faces more rapidly when presented together with their own smell than with that of someone else. Another kind of semantic priming has been shown to occur when the gender that is associated with a scent is used to prime, or modulate, the perception of sequentially/simultaneously presented gender-matched, as compared to gender-mismatched, faces.

In one of the first studies of this type, Capparuccini et al. (2010) sprayed an experimental room with one of *Givenchy’s* male or female perfumes (*Pi Neo* or *Angel or Demon*, respectively) as the olfactory stimulus during visual assessments of a range of 10 attributes/qualities using 10-point Likert scales. The participants (male and female) had to rank five male and five female faces against the (neutral, non-sexual) adjectives of ‘familiarity’ and ‘confidence’, (the potentially less neutral adjectives of) ‘liking’ and ‘irritability’, (and the putatively more sexually pertinent adjectives of) ‘beauty’, ‘pleasantness’, ‘charm’, ‘intensity’, ‘sexual interest’, and ‘sexual attraction’. The fragrance was sprayed in the room prior to the participant’s arrival, and (once again) no explicit mention of its presence was made by the experimenters. Of particular interest to the experimenters in this case was the change in their participants’ ratings of the face between the experimental sessions in which the scent happened to be congruent versus incongruent with the sex of the participant that they had to rate. Rather surprisingly, it turned out that gender-congruency of the faces being rated didn’t appear to influence the pattern of results that were obtained.

Intriguingly, judgments of beauty and charm were both enhanced by the presence of a gender-congruent (with the participant) as compared to a gender-incongruent ambient fragrance, with beauty showing the largest effect. These results were taken to show that the use of sexually oriented perfumes (i.e., gender-congruent, though in this case, as we have just seen, the congruency was relative to the participant rather than the face being rated) can have relatively large effects on the judgment of the more hedonic (or affective) aspects of person perception. By contrast, the gender-congruency of the ambient fragrance had less of an influence on the more purely sexual judgments, and no effect whatsoever on the neutral non-sexual ratings of familiarity and confidence. Once again, though, with only one example of gender-congruent and gender-incongruent scent it is impossible to know whether similar crossmodal effects would necessarily have been documented were other male and female fragrances to have been used instead.

Elsewhere, Marinova and Moss (2014) reported that people’s ratings of various characteristics of person perception beyond just attractiveness or pleasantness were affected by the presence versus absence of gender-congruent versus gender-incongruent fragrance. The female participants who took part in this study had to rate 15 male faces (five of high attractiveness, five of medium attractiveness, and five of low attractiveness) on six attributes: attractive, reliable, outgoing, intelligent, wealthy, and socially competent. Ratings were made in the presence of a female perfume (incongruent condition), a male perfume (congruent condition), or a no perfume control condition, with participants randomly allocated to produce three groups of equal size (i.e., a between-participants experimental design was used). Spraying a room with four spritzes of fine fragrance prior to each participant’s arrival presumably created a somewhat ambiguous situation in terms of whether the scent was perceived by participants to be an ambient fragrance or else someone’s personal fragrance (cf. Pichon et al., 2015).

The results failed to reveal a main effect of perfume congruency on attractiveness ratings. However, the moderately attractive male faces were rated as significantly more attractive by those in the gender-congruent as compared to the gender-incongruent condition, thus giving rise to an interaction between perfume condition and attractiveness group. Nevertheless, those participants exposed to the gender-congruent fragrance still rated four out of the five so-called ‘halo’^11^ characteristics (namely, ‘outgoing’, ‘intelligent’, ‘wealthy’, and ‘socially competent’) more highly than those in at least one of the other two conditions. So, for example, those faces presented while sniffing the gender-congruent scent were rated as looking significantly more intelligent than when viewed in the absence of fragrance (the difference with the gender-incongruent fragrance in this case failing to reach statistical significance). These results therefore indicate that the presence of an ambient gender-congruent perfume can impact positively on first impressions beyond attractiveness. However, it is worth noting that the small sample size, coupled with the between-participants nature of the fragrance manipulation, likely limits the power of this particular study. Furthermore, the use of only one gender-congruent and one gender-incongruent fragrance again means that it is simply not possible to disentangle whether it is the perfume, or the gender-congruency with the faces, that is doing the work in terms of influencing participants’ ratings. In conclusion, given the lack of a main effect of olfaction on attractiveness, and given the between-participants manipulation of the olfactory stimulus, Marinova and Moss’s (2014) results might well be considered more likely to reflect olfaction’s effect on mood (cf. Kirk-Smith & Booth, 1990), rather than a direct crossmodal effect on attraction, leading to a halo effect that then carried over to influence other judgments about a person.

Finally, in a recently published study, Risso et al. (2021) had their participants rate the attractiveness of male and female faces presented on a monitor (on a VAS anchored with the terms ‘unattractive’ and ‘attractive’) while in the presence of no odour (air), a liquorice odour, or a caramel odour. These two food odours had been rated as more masculine or feminine, respectively, in a preliminary study with 12 food aromas.^12^ In total, each one of 60 faces was paired once with each of the three odours. The odorants were presented from glass bottles, with participants instructed to sniff while viewing and rating the face on the screen. Once again, the participants were told nothing about the link between these suprathreshold food-related odours and the faces they saw. Nevertheless, the male faces were still rated as looking significantly less attractive in the presence of either odour (with a slightly, but significantly, bigger drop for caramel, whereas for the female faces, the only significant drop in attractiveness ratings occurred while sniffing the liquorice as compared to the caramel aroma, with ratings in the latter condition being no different from the no odour control.^13^

Risso et al. (2021, p. 1) interpreted their results as highlighting: ‘the importance of the synaesthetic associations between “gender” and odours on people's judgements of facial attractiveness’. While the notion of crossmodal correspondences probably fits better with the contemporary view of the consensual link that so clearly exists between gender and non-body-related scents (Spence, 2011; Zellner et al., 2008), one might wonder whether this requires an additional explanation for the crossmodal effects of olfaction on person perception over-and-above those that have been outlined thus far. Perhaps the simplest way to think about these results is in terms of crossmodal semantic priming in this case based on the gender that was associated with the scent (though see also Lindqvist, 2012a, 2012b)*.*^14^

### Olfactory contributions to the perception of facial emotion

Beyond the effect of hedonically valenced or gendered olfactory stimuli on judgments of the attractiveness of faces (and, on occasion, other personal attributes), a separate line of experimental research has shown that hedonically valenced non-body-related odours can modulate (that is, either facilitate or impair) the speeded identification of facial emotion too (e.g., see Leppänen & Hietanen, 2003; Seubert et al., 2010a). So, for example, Leppänen and Hietanen reported that their participants were able to recognize disgusted facial expressions more rapidly in an unpleasant odour context while happy expressions were recognized more rapidly in a pleasant odour context instead. The presence of pleasant versus unpleasant odours have also been shown to result in the enhanced recognition of both disgusted and happy facial expressions (Seubert et al., 2010b; see also Li et al., 2020; Stankovic et al., 2020, who presented isovaleric acid). Using functional MRI, Seubert et al. (2010a) were able to demonstrate that the processing of disgusted faces was facilitated by the prior presentation of odour primes. In the latter study, non-body-related pleasant (vanillin) and unpleasant odours (H_2_S) were presented, and compared to a no odour baseline condition. However, regardless of the valence of the olfactory stimulus, a reaction time advantage (olfactory priming) was documented for the recognition of disgust, but not for the recognition of either happy or neutral faces.

Elsewhere in the literature, establishing the appropriate odour-induced context (positive strawberry, vanilla, and orange zest, vs. negatively valenced fish odour) has been shown to modulate the search advantage for happy facial expressions amongst neutral faces (Damjanovic et al., 2018). Other researchers, meanwhile, have highlighted how odour-evoked hedonic contexts influence the discrimination of facial expressions in the human brain (Kastner et al., 2016; Poncet et al., 2021). In the latter research, contextual valenced odours influenced the discrimination of a neutral face, and to a lesser extent of a face showing disgust, as indexed by an occipito-temporal facial expression-specific brain response. In particular, the neural response to the neutral faces was found to be respectively larger and lower in the context of pleasant and unpleasant odours as compared with the control odour.

According to research by Forscher and Li (2012), micro-fearful facial expressions are processed preferentially following olfactory priming by semantically unrelated negative odours (valeric acid—sweat/rotten cheese and hexanoic acid—rotten meat/fat) as compared to more neutral olfactory stimuli (grass/medicine and pine resin scents). Setting a congruent odour context has also been shown to facilitate the perception of low-intensity emotional facial expressions (Leleu et al., 2015a, 2015b). That is, a congruent odour can help to reduce the amount of information that a participant needs to recognize a congruent emotional facial expression.

One final preregistered study that is worth mentioning here was reported by Syrjänen et al. (2017). These researchers observed that valenced odors exerted a much reduced influence over the evaluation of emotion in dynamic (i.e., rather than static) faces. As in another study from the same research group that was mentioned earlier (Novak et al., 2015), the dynamic facial stimuli morphed from neutral to emotionally expressive. The participants had to classify a series of dynamic facial expressions as happy or disgusted. Syrjänen and colleagues wanted to know whether the emotional evaluation of these facial expressions would be affected by exposure to a negatively valenced sweat-like, odour (valeric acid—sweat), as compared with a soap-like, positively valenced odour (lilac essence), or a no-odour control. However, the results revealed that the pleasant and unpleasant odours had no effect on the time needed by participants to recognize happy or disgusted dynamic facial expressions.^15^ It is, though, perhaps somewhat unclear as to whether this null result should be attributed to dynamic nature of face stimuli, or else to the fact that these faces both started with the same neutral emotional expression. Furthermore, it can also be wondered whether the use of a blocked design, where the participants were exposed to each odour over a five minute block of trials (i.e., rather than a trial-by-trial design) might not also have contributed to the null results. It is perhaps also worth noting that performance in this task was quite high (*d'* 2.5 for disgusted, and a *d′* of a little over 3 for happy expressions). The latter observation is important inasmuch as it has been suggested, and, in fact, demonstrated that olfactory stimuli may have a more pronounced influence over visual perception under those conditions where the visual task is more ambiguous/difficult (e.g., see Forscher & Li, 2012; Mujica-Parodi et al., 2009; Novak et al., 2015; Rubin et al., 2012; Zhou & Chen, 2009; see also Leleu et al., 2020).

Some researchers have even argued for a superadditive brain response, based on inverse effectiveness, in response to the simultaneous presentation of subtle visual (facial) and olfactory cues linked to negative emotion (such as faintly fearful faces, and the negatively valenced odours described as smelling of ‘rotten fish’, ‘sweat/rotten cheese’, ‘rotten meat/fat’, ‘rotten egg’ when presented at a suprathreshold level, but here presented at a near-threshold level.^16^ Taken together, the results that have been summarized in this section would appear to fit in a framework in which hedonically valenced contextual olfactory stimuli facilitate the processing of congruent (in terms of hedonics) facial expressions. This is evidenced behaviourally as an enhanced ability to process facial emotion. Neurally, the evidence further indicates that emotionally charged odours modulate visual cortical response to ensuing emotional faces (Forscher & Li, 2012; Seubert et al., 2010a).

### Are the effect of olfaction person perception bidirectional?

Before concluding this section of the review, it is perhaps worth considering the question of whether crossmodal/multisensory influences also operate in the reverse direction. It is certainly noticeable how the vast majority of the literature on multisensory person perception has tended to focus solely on any crossmodal influences of olfactory cues on visual assessments rather than vice versa. What is more, this asymmetry becomes all the more striking when considered in the context of the separate literature that has emerged over the last half century or so concerning the crossmodal correspondences that exist between odour and colour. In the latter case, virtually all of the research that has been published to date has tended to focus on those crossmodal influences operating in the opposite direction: Namely, researchers have almost exclusively focused on the influence of vision (colour) on olfaction, rather than vice versa (see Spence, 2020e, for a recent review).

One of the few exceptions to this general asymmetry in the literature on multisensory person perception comes from a study by Cook et al. (2017) in which the pleasant fragrance of jasmine was rated as smelling significantly more pleasant when participants were staring at a happy rather than at a disgusted face. Meanwhile, participants rated the negatively valenced odour of rotting cabbage as smelling more intense when staring at a disgusted as compared to a happy face (once again, note that the odours are not obviously person-related; cf. Hummel et al., 2017). That said, it should be stressed that the crossmodal effects of vision on olfaction were pretty small in magnitude in this case. Elsewhere, Incollingo Rodriguez et al. (2015) reported on a couple of studies in which their participants viewed images of heavy (i.e., overweight/obese) and normal weight individuals while smelling coloured substances that, unbeknownst to them, were actually odourless. Across the two studies, the olfactory stimuli were rated as smelling worse when they were paired with images of heavy individuals than when they were paired with images of thin individuals. Once again, therefore, such results hint at the possibility that crossmodal influences may sometimes be observed from visual cues (of faces or bodies) on olfactory judgments, even when no explicit link is made by the experimenters between the olfactory stimuli and the people shown on screen.

### Interim summary

The results that have been reviewed in this section clearly reveal that hedonically valenced odours, as well as semantically meaningful odours (typically gender-congruent vs. gender-incongruent) can, and very often do, influence person perception. Both hedonically valenced, and gendered fragrances have been shown to influence ratings of the people shown in still, if not necessarily in dynamically changing, images (see Novak et al., 2015; Syrjänen et al., 2017). Meanwhile, combining the results reported by Capparuccini et al. (2010), Marinova and Moss (2014), and Seubert et al. (2014), it would appear that the crossmodal effects of olfaction on visual perception are typically more apparent for certain judgments (attributes) than for others. In particular, crossmodal effects on visual ratings appear most pronounced for judgments of facial attractiveness (considered part of the affective system and perhaps indexing mate-selection; see also Corley & Raudenbush, 2002) and the seemingly interchangeably used terms of pleasantness (Cook et al., 2015, 2017, 2018), likability (Li et al., 2007), and beauty (Capparuccini et al., 2010).^17^ The evidence is much weaker for any crossmodal influences of hedonically valenced odours on the more neutral, or cognitively determined attributes of faces, for example, judgments of well-being (Rotton, 1983) or age (Seubert et al., 2014). And the limited research that has been published to date has failed to demonstrate any impact whatsoever of olfactory stimuli on judgments of neutral attributes such as ‘energy’ (Rotton, 1983), ‘familiarity’, or ‘confidence’ (Capparuccini et al., 2010). At the same time, however, a separate line of experimental research has repeatedly demonstrated how the presentation of hedonically valenced olfactory stimuli frequently do influence the perception (i.e., the detection/discrimination) of the emotion shown by faces too. The latter crossmodal effects typically, but not always, being shown when the hedonic valence of the olfactory stimuli was congruent with the to-be-judged facial emotion (e.g., a hedonically negative odorant with a disgusted face). And, as mentioned earlier, one other important factor that appears to modulate the influence of olfactory cues on visual person judgments is the difficulty, or ambiguity, of the visual task.

Thus, taken together, the evidence reported so far in this narrative review would appear to show that olfactory stimuli can (but by no means always do) influence visual ratings of other people (see Table 1). A priori, one might have expected to observe either crossmodal ‘assimilation’ or ‘contrast’ effects in visual person judgments following the presentation of a task-irrelevant olfactory prime (e.g., Deliza & MacFie, 1997; Li et al., 2007; Piqueras-Fiszman & Spence, 2015). It is interesting to note, therefore, that the vast majority of the studies reported in this section have documented that when a crossmodal effect is evidenced assimilation is nearly always the result (e.g., Demattè et al., 2007; Marinova & Moss, 2014; Risso et al., 2021).

Over the years, several distinct cognitive mechanisms have been put forward in order to try and account for the crossmodal influence of task-irrelevant olfactory stimuli on visual person judgments. In those early studies where the olfactory stimulus was manipulated on a block-by-block, or between-participants, basis, and thus where the participants were exposed to a particular ambient odour for longer (than in the case in trial-by-trial priming studies), the indirect consequences of a particular mood being induced as a result of exposure to the hedonically valenced odour has been suggested to be behind the changed ratings. However, in the majority of more recent studies, where the olfactory stimulus has been changed on a trial-by-trial basis, and has typically been presented prior to the visual stimulus, then crossmodal affective (in the case of hedonically contrasting odours), or semantic (in the case of gender-congruent versus gender-incongruent odorants) priming has been suggested as the most likely mechanism underpinning the crossmodal effects that have been reported (see Table 2).

**Table 2:** Summary of the most popular explanations that have been put forward to explain the crossmodal influence of olfaction on visual judgments of a person's facial attractiveness (and other attributes of person perception

Mood-induced changes	Rotton (1983) and Kirk-Smith and Booth (1990)
People look better/worse when we are in a good/bad mood	
Crossmodal affective priming	Demattè et al. (2007), Li et al. (2007) and Cook et al. (2015, 2018)
The valence associated with an olfactory prime can bias people's judgment of the attractiveness/likeability of a subsequently presented visual face	
Crossmodal semantic priming	Hirsch (2006)
A familiar scent may be associated with someone of a certain age, and this form of semantic priming may bias judgments of a person's age	
Crossmodal gender congruence	Capparuccini et al. (2010), Marinova and Moss (2014) and Risso et al. (2021)
The presentation of a gender-congruent (pleasant) fragrance may sometimes boost attractiveness, as a result of semantic priming and/or based on crossmodal correspondences	
Halo-dumping	Demattè et al. (2007)
People may rate photos as more attractive in presence of pleasant olfactory stimulus only because they have no way to indicate what they think about the olfactory stimulus. (This account ultimately rejected.)	
Olfactorily induced change in arousal	Bensafi et al. (2002), Hirsch (2006) and Risso et al. (2021)
Olfactory stimuli may influence a person's level of (sexual) arousal, and this, in turn, may influence their rating of other people	

However, rather than seeing these different explanations as being entirely mutually exclusive, it is perhaps worth highlighting the fact that both the affective priming and mood induction accounts involve an emotional response to smell, differing primarily in the timecourse of the crossmodal effects (or emotional response). Meanwhile, the crossmodal affective, semantic, and gender-based priming accounts are similar inasmuch as they all stress the (in)congruency of the signals presented to the two modalities, vision and olfaction.

At the same time, however, it is perhaps worth highlighting one other potential explanation for the crossmodal effects of fragrance, namely ‘halo dumping’, that was raised, although ultimately discounted, by Demattè et al. (2007). The notion of halo dumping first emerged out of research on olfactory–gustatory interactions (e.g., in fruitiness/sweetness perception) where it was argued that the failure to provide appropriate response alternatives (in this case concerning sweetness) can lead participants to ‘dump’ what they think about an easy to rate attribute, namely sweetness on the fruitiness rating scale, since that was the only that had been provided to them to express themselves (Clark & Lawless, 1994). In this case, providing a separate scale for participants to respond to both gustatory sweetness and olfactory fruitiness, eliminated the crossmodal effect of sweetness on participants’ fruitiness ratings. One might consider whether the few studies of the olfactory modulation of person perception where the participants have been given a number of different scales to rate a person’s attributes (six in the case of Marinova & Moss, 2014; ten in the case of Capparuccini et al., 2010; and two in the case of Seubert et al., 2014), presumably help to address this potential concern, as do McGlone et al.’s (2013) neuroimaging results (discussed earlier).^18^ At the same time, however, it is also worth noting that the senses of taste and smell are much more closely connected (and hence easily confusable; Spence, 2015, 2016) than are olfaction and vision.^19^ This may perhaps reduce the likelihood of this potential alternative explanation for the data (though see also Kappes et al., 2006). Given that the halo dumping account has not reappeared in the literature since first being discounted by Demattè et al. (2007), it can presumably be ignored as a relevant potential explanation for the crossmodal effects under study.

What is far less clear, at least on the basis of the studies that have been reported thus far in this review is the extent to which the nature of the relationship between the odour and the people being judged affects the likelihood of significant crossmodal effects being observed. A priori, given the existing literature on multisensory integration, one might legitimately have expected the likelihood of observing crossmodal effects to vary as a function of the spatial, temporal, and/or semantic relation between the unimodal stimuli (e.g., Calvert et al., 2004). However, what is particularly striking, looking back over all of the studies that have been reviewed here, is how the experimenters have rarely, if ever, made mention of the fragrance(s) or what its/their role, or source, might be (e.g., Bensafi et al., 2002). As such, one would have thought that the participants would have had little reason to bind the visual stimuli shown on the computer, and hence not expected to smell, with the odour that was present in the room, in a bottle (Risso et al., 2021), or more commonly these days, delivered by means of an olfactometer. That crossmodal effects have so often been documented then perhaps hints at the ubiquity/robustness of such effects and their seeming insensitivity to the normal rules of multisensory integration, at least as they have been reported amongst the spatial senses.

## Limitations/peculiarities of crossmodal research on olfaction on person perception

In this section, I would like to highlight some of the peculiarities, and hence possibly also limitations, of the majority of the crossmodal research on fragrance effect on various aspects of person perception reported in the previous section. A slightly more subtle distinction here is between the suggestion that spatiotemporal coincidence is simply less important for olfactory multisensory integration, and the claim that it would be important were such information to be available in the olfactory system, which it mostly is not (see Sela & Sobel, 2010).

### Does familiarity matter?

The first thing to note is that unfamiliar faces have nearly always been used as stimuli. It is certainly possible that the presence of a given fragrance might be more likely to exert a crossmodal influence over judgments of those people whom we are unfamiliar with, rather than those whom we already know well (and hence have perhaps made up our mind about; Graham & Jouhar, 1980). Indeed, elsewhere in the world of multisensory research, it has, on occasion at least, been demonstrated that audiovisual interactions (namely the McGurk effect when elicited by gender-incongruent auditory and visual speech stimuli) is modulated by the participants’ familiarity with the person shown/heard in the stimuli that they are judging (see Walker et al., 1995; cf. Setti & Chan, 2011). Hence, in real-world interactions with those whom we are familiar, it might be assumed that ambient malodour, or personal scent, would exert less of an influence than might be suggested by much of the literature reviewed here. As yet, though, this question has not been addressed empirically.

### What is the difference between pictures and real people?

Given that we do not expect static photos or even dynamic videos to smell, it is perhaps surprising that so many significant effects have been reported using such discrete unisensory stimuli. At the same time, however, one might have imagined that if we had to evaluate an actual person wearing a fragrance, then perhaps any effects of olfaction on person perception ought to be enhanced (given that real people do smell). As far as I am aware, the only study to have done something along these lines was reported almost two decades ago by Sczesny and Stahlberg (2002; Experiment 2). These researchers conducted a social psychology experiment in which the male or female participants (*N* = 116 in total) had to pretend to be a personnel manager conducting a job interview of a male or female confederate who entered the room wearing a typical male or female fragrance (the most extreme of 12 fragrances evaluated in a pre-test). A significantly higher number of the candidates wearing the male fragrance were offered a job than were those wearing either no perfume or else a female fragrance. It might have been expected that a violation of odour expectations would have been triggered by those wearing a gender-incongruent scent. This, in turn, might then have captured the participants’ attention thus eliciting a heightened evaluative response (either positive or negative, depending on the valence of the odour; Schneider et al., 1979). However, in the case of Sczesny and Stahlberg’s study, the gender-congruency of the scent did not much seem to matter. That said, it is hard to say any more about this particular case given that the identity of the fragrances used was never revealed. Once again, empirical evidence concerning the relative influence of scent on our ratings of those who are either physically present in front of us, or else merely depicted in a photo on a computer screen is also currently lacking (presumably, in-part, due to the difficulty of conducting research with real people).^20^

### How important is the use of static versus dynamic images

It is striking how all but one of the 16 studies reported in Table 1 used static images of people’s faces. Reasons to believe that this feature of the experimental stimuli used in the majority of the research in this area shouldn’t much matter comes from those findings showing that judgments of facial attractiveness tend to be highly correlated for static photos versus dynamic video clips (Roberts et al., 2009a, 2009b). At the same time, however, the crossmodal research that has been published to date appears to suggest that olfactory cues exert much less of an influence over judgments of dynamic visual stimuli (e.g., morphing faces, Novak et al., 2015; Syrjänen et al., 2017), perhaps because the latter are more likely to capture a viewer’s attention (Krumhuber et al., 2013; Sato & Yoshikawa, 2007). Hence, given that in real life face-to-face encounters we normally do have access to dynamic facial cues, one might worry how much of an impact ambient or personal fragrance is really going to have.

### How important is it that olfaction is nearly always presented first?

Another area where additional crossmodal research might be considered beneficial to developing a better understanding of the parameters influencing the crossmodal influences on person perception links to the role of the relative timing of olfactory and visual cues on the behavioural and neural effects that may be observed. In other areas of crossmodal priming research, useful information is often provided by assessing the timecourse of presentation of one stimulus on the perception of, or response to, another more or less closely related stimulus (e.g., Chen & Spence, 2013; Smeets & Dijksterhuis, 2014; Wang et al., 2020). What is noticeable about the majority of studies of olfaction’s crossmodal influence over visual judgments of people is how the onset of the olfactory stimulus nearly always precedes that of the to-be-judged visual face image (though see Cook et al., 2018). To the extent that the ordering of unisensory impressions is likely to be reversed in everyday life, one might again wonder just how relevant the tightly constrained laboratory research is to the kinds of crossmodal or multisensory interactions that are likely to be seen in a more ecologically valid real-life setting. At the same time, however, it is also important to bear in mind that sensory transduction of olfactory stimuli at the nasal epithelium takes several hundred milliseconds longer than the transduction of visual stimuli at the retina (see Spence & Squire, 2003).

It is perhaps relevant here to note that we typically make up our minds concerning a person within the first 100 ms of seeing them (Willis & Todorov, 2006), with ratings of attractiveness, likeability, trustworthiness, competence, and aggressiveness all being highly correlated with the judgments we make when not under time constraints. Survival-related judgments of threat from facial stimuli can be made based on whatever visual information is available during the first 39 ms of seeing someone (Bar et al., 2006), while their facial attractiveness can be estimated in nothing more than a glance (Olsen & Marshuetz, 2005). According to Carbon and colleagues, we can identify someone’s gender from a portrait after 244 ms, and rate their attractiveness just 59 ms later (Carbon et al., 2018; Dobson, 2018). Given the rapid processing of visual information, and the much slower processing of olfactory stimuli, one might therefore wonder whether this means that our mind has normally been made up on seeing someone for the first time prior to any olfactory cues generally becoming available and hence influencing our judgments subsequently, or retrospectively (see also Walla, 2008)? This is where further crossmodal priming studies in which the stimulus onset asynchrony (SOA) between olfaction and vision is parametrically varied might be especially useful. The results of such research might then help to determine the ecological validity of crossmodal olfactory effects on visual judgments of people.

### What role does scent attribution play?

There is an important question concerning the attribution of olfactory stimuli that runs through all of the research that has been reported thus far in this review. In particular, in none of the studies that were mentioned in the preceding section did the experimenter ever give their participants an explicit reason to link the odours to the unknown people whose images they were being asked to judge. As such, given how the majority of studies reported a significant crossmodal effect of olfaction on visual person judgments, one might be tempted to conclude that olfactory stimuli are automatically integrated with whichever other (visual) stimuli happen to be presented, or attended, at the time. The correlated delivery of scent and picture in many of the studies would suggest that approximate temporal coincidence certainly provides sufficient reason to bind (Cook et al., 2018). Of course, the claim that olfactory stimuli are simply bound with any visual stimuli that happen to be presented at around the same time, is certainly much harder to accept in the case of visual search studies, say, where multiple faces may be presented at the same time (see Damjanovic et al., 2018). This presumably giving rise to one version of what might perhaps be considered the olfactory version of the cocktail party effect (see Rokni et al., 2014, for another).

It is, then, rather surprising that the attribution of the odour does not seem to matter in this case. The question of what, if anything, a participant attributes the scent’s presence to is simply left unmentioned. However, several possible alternatives immediately suggest themselves—people might attribute the odour to the environment (Rotton et al., 1978; see Spence, 2020c), to the person that they happen to be evaluating at that moment (Demattè et al., 2007), or perhaps to the clothes that they happen to be wearing (Demattè et al., 2006; Laird, 1932).^21^ Alternatively, however, the research also shows that olfactory cues may influence the perceived shininess of a person’s hair (Churchill et al., 2009), or even the product that they happen to be inspecting (Aikman, 1951; Ebster & Kirk-Smith, 2005)? Intriguingly, the question of how to ensure the appropriate attribution of scent also arises in the case where film-makers have attempted to link particular scents, or fragrances to individual characters in the case of scented cinema; see Spence, 2020d, for a review). In one conference abstract relevant to this theme, Wille et al. (2003) had people watch videos while in the presence of a pleasant odour (including a jasmine-like fragrance), an unpleasant odour (indole), or else no odour. The presence of either odorant influenced the participants’ mental state while at the same time also leading to their rating the actors as looking more attractive. This is a somewhat counterintuitive result in the case of the negatively valenced smell of indole, though it is worth noting that, when questioned, the participants did not want to see more of the movie clips in that condition.

Given the lack of spatial information typically provided by our olfactory experiences (meaning that the location of the source of an olfactory stimulus is normally unavailable within the olfactory system), temporal coincidence, combined with semantic knowledge would appear much more important to determining what is bound with, or influenced by, a given scent (though see also Sela & Sobel, 2010, for the suggestion that humans are in a constant state of olfactory change blindness). One other thing to note is that the rise of so-called gourmand fragrances—that is, fragrances that have a distinctly flavourful olfactory expression might be expected to make it somewhat harder for the perceiver/participant to attribute the fragrance to the correct source, given the more obvious semantic association with a real-world food source (see also Anon, 2008; Barr, 2020; Tanner, 2017). That is, it is unclear why exactly the smell of liquorice or caramel should have been bound with, or at least have influenced attractiveness ratings in Risso et al.’s (2021) study.^22^

## The influence of a person’s natural body odour on multisensory person perception

All of the research that has been reviewed thus far in this narrative review has relied on the presentation of non-body-related scents with the closest thing to natural body odours being the synthetic BO (thiol compound), used in the research by Demattè et al. (2007) and McGlone et al. (2013), and the synthetic sweat (valeric acid) used as a negatively valenced stimulus in the studies reported by Li et al. (2007), Forscher and Li (2012), and Syrjänen et al. (2017). However, a large body of research now shows that we are able to make approximate judgments about an individual based on nothing more than a brief exposure to their natural scent.^23^ So, for example, as was mentioned in the Introduction, we can estimate a person’s rough age (Mitro et al., 2012), their sex (Schleidt et al., 1981), their health and emotional state (Chen & Haviland-Jones, 2000; including chemosensory signatures of fear and anxiety; de Groot & Smeets, 2017), and make informed guesses about three of the big five personality traits at a level that turns out to be significantly better than chance (Sorokowska et al., 2012, 2016). We can even smell out those who are related to us (i.e., our kin; Weisfeld et al., 2003). In this section, I would like to briefly review the research that has been published on the multisensory integration of various body odours with visual judgments concerning person perception. Note that, in this case, it feels somewhat more natural to talk in terms of multisensory integration, rather than simply just crossmodal effects, given that every one of us has our own body odour (i.e., the various cues could potentially belong to the same person/object).

### Chemosensory signals of anxiety and stress

The presence of sweat odour has been shown to increase the rated arousal of faces, and may also enhance the early allocation of attentional processes in the structural encoding of faces (Adolph et al., 2013). Mujica-Parodi et al. (2009) observed increased amygdala activation following the presentation of chemosensory stress stimuli. Indeed, threatening or fearful stimuli (such as fearful faces and stressed body odours activate the brain’s fear circuit including the amygdala (Novak et al., 2015; Rocha et al., 2018; Zald & Pardo, 1997). Rubin et al. (2012) reported that inhaling stress sweat enhances the neural response to neutral faces. Walla et al. (2003) used magnetoencephalography (MEG) to study olfaction and face encoding in humans.

Elsewhere, it has been shown that the positive emotional priming of the perception of facial affect in females is diminished in the presence of chemosensory anxiety signals (Pause et al., 2004). Specifically, the presence of chemosensory anxiety signals collected from men awaiting an exam was shown to reduce the priming effect of a briefly presented happy face presented prior to a neutral face (rated for facial affect) in women, but not in males, The presentation of stress chemosignals have also been shown to influence social judgments of people (women) shown in videos (Dalton et al., 2013). In particular, the women shown in video scenes were rated as being more stressed by both men and women when in the presence of stress sweat. The male participants also rated the women in the videos as looking less confident, trustworthy and competent when smelling the stress or exercise sweat (as compared to deodorized stress sweat condition).

Rocha et al. (2018) demonstrated that the presentation of BO sampled from individuals who were anxious induces a stress response and, at the same time, can bias the recognition of dynamic facial expressions, when compared with the BO taken from relaxed individuals. The participants (*N* = 46) had to categorize the emotion of a face that morphed from a neutral expression to either an angry or happy expression, during exposure to either stressed or relaxed BO. Exposure to the anxiety BO increased the accuracy of dynamic facial recognition (while, at the same time, reducing parasympathetic cardiac activity). These results therefore suggest that those components of BO that are associated with anxiety induce a stress response in recipients, modulating both their arousal and cognitive-emotional skills but at the same time facilitating the processing of emotional facial stimuli (cf. Mutic et al., 2019). The presence of a fear odour facilitates the detection of fearful expressions over other negative expressions (Kamiloglu et al., 2018; cf. Jessen, 2020). Meanwhile, Zhou and Chen (2009) found that the chemosignal of fearful sweat biased women toward interpreting ambiguous facial expressions as more fearful, but has no effect when the facial emotion was more obvious (see also Prehn-Kristensen et al., 2009).

### Non-fear/anxiety related chemosensory body odours

The presence of human sex hormone-like chemicals also influence people’s ratings of the perceived masculinity/femininity of the faces shown on the computer (see Kovács et al., 2004; see also Zhou et al., 2014). A single intranasal dose of the neuropeptide oxytocin (involved in attachment behaviours) has been shown to enhance the facial judgements of attractiveness and trustworthiness with respect to an intranasal dose of placebo (Theodouridou et al., 2009). Meanwhile, Striepens et al. (2014) found that oxytocin enhances the perceived attractiveness of unfamiliar female faces.

### Conditioned responses to body odours

At this point, it is perhaps worth drawing attention to the fact that people develop conditioned associations with the body odours of attachment figures—think here only of the calming scent of a loved one (Granqvist et al., 2018; Hofer et al., 2018; McBurney et al., 2006, 2012; Shoup et al., 2008; though see also Black, 2001). There is also growing evidence of the importance of maternal odour to the perception of faces in newborns (Leleu et al., 2020; Rekow et al., 2020). Meanwhile, some years ago now, Sullivan and Toubas (1998) described the positive effect that maternal odour had on soothing (i.e., reducing crying) and feeding preparatory responses (i.e., mouthing) in newborn (breast- and bottle-fed) babies.

In adults, such crossmodal effects are presumably based on an individual’s prior emotional experiences (see also Janssens & De Pelsmacker, 2009). Though, in such cases, it may be difficult to rule out any influence of the intrinsic qualities of the body odour from the positive associations that have subsequently been conditioned through extensive repeated exposure to the odour. In the future, it would be interesting to know whether a fine fragrance that was robustly attached to a loved one (should they always choose to wear the same scent) might take on some, or even all, of the same-stress reducing properties as body odours, or whether instead there is something special about the latter (see Boyle et al., 2009; Lundstrom et al., 2008; though see also Cecchetto et al., 2020).

### On the surprising relationship between body odour and fragrance choice

**Fig. 5:**
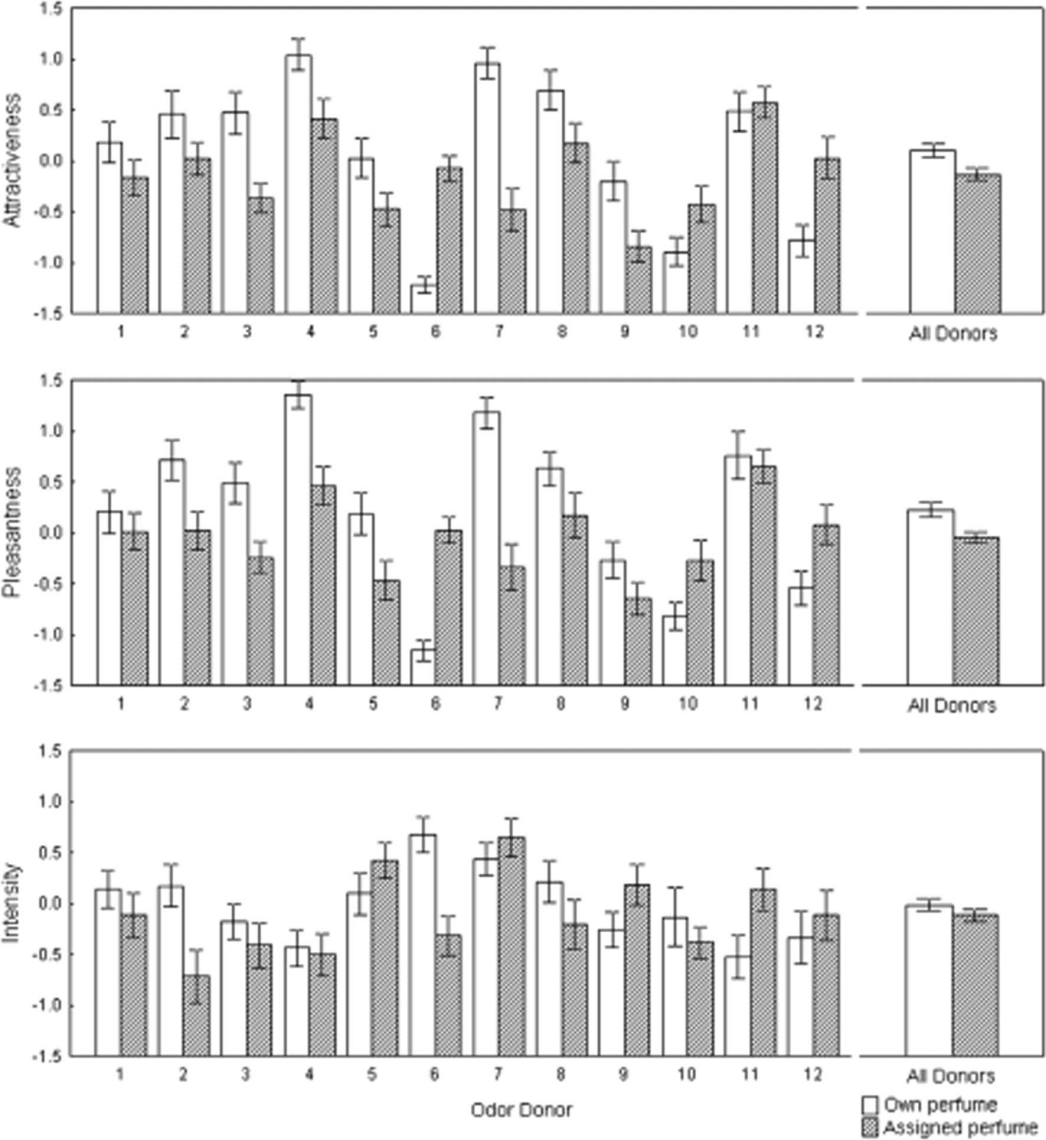
Ratings of own and assigned perfume-body odour blends in Lenochová et al. (2012, Study 3). *Z*-scored mean ratings (6 SEM) of attractiveness, pleasantness and intensity of perfume-body odour blends in individual male odour donors and for all donors together. Empty bars signify own and preferred perfume while shaded bars represent randomly assigned perfume combined with donor’s individual body odour. [Figure reprinted with permission from Lenchová et al. (2012, Figure 4). 10.1371/journal.pone.0033810.g004.]

In many parts of the world, people choose to mask our personal odours (Schleidt et al., 1981; see also Ferdenzi et al., 2013; König, 1972). That being said, there is an intriguing emerging literature on the non-random nature of the fragrance choices that we make, and how they may actually serve to, in some sense, amplify the olfactory signals that we give off naturally (see also Veitinger, 2015).^24^ So, for example, Allen et al. (2016) demonstrated that the rated femininity of body odour was enhanced by the use of fragrance in women (though not in men). Milinski and Wedekind (2001) reported evidence of major histocompatibility complex (MHC)-correlated perfume preferences in people (see also Hämmerli et al., 2012). Meanwhile, Lenochová et al. (2012) found that the mixture of a person’s body odour with their preferred choice of perfume (taken from 12 young male adult donors) was rated as significantly more pleasant by a group of 21 young female adult assessors than when the male donor’s body odour was mixed with another (equally pleasant) fragrance chosen (randomly allocated) by the experimenter instead (see Fig. 5). Hence, while personal fragrance is undoubtedly an artificial creation, it may come to take on at least some of the role of authentic body odours (in providing an attractive olfactory signal), should it be experienced on an individual sufficiently often (see also Behan et al., 2006).

### Interim summary

Body odours provide a rich source of information (albeit often perceived unconsciously; Lübke & Pause, 2015; Parma et al., 2017; Pause, 2012) about a number of personal qualities.^25^ Given that they constitute an intrinsic part of a person’s make-up, combining them with other attributes of person perception should perhaps best be considered as an example of multisensory integration (i.e., rather than as a crossmodal effect). At the same time, however, it is important to note that the body odours used in the majority of studies have been taken from other individuals than those assessed visually. This means that while the odour might be construed as, in some sense, congruent, it did not originate from the same person/object. Body-related chemosensory stimulation can, though, sometimes interfere with the visual information processing of faces (cf. Walla et al., 2005). And, more generally, it has been reported that odour perception can interfere with verbal processing (and vice versa; see Lorig, 1992; Walla, 2008; Walla et al., 2003; see also Zhou et al., 2019).

## Olfactory influence on perception of the self

While the majority of the research that has been published to date has tended to focus on the effect of scent/odour on the perceiver’s perception of other people, there is also likely an impact (especially of one’s own body odour, or scent) on self-perception too, in terms of relaxation, self-confidence, mood, and/or arousal (Graham et al., 2000; see also Eli et al., 2000). At the same time, however, it is worth noting for how long (historically speaking), and how frequently (especially nowadays), people have been scenting themselves. According to Stoddart (1990), the use of perfumes and fragrances dates back at least as far as the ancient Egyptians and Greeks. In one UK study, 79% of the women and 60% of the men sampled reported using a deodorant every day (Roberts et al., 2010). People use fragrance and deodorize themselves for various reasons, including everything from the avoidance of being stigmatized through to enhancing the sense of personal appeal and confidence (Freyberg & Ahren, 2011; Waskul & Vannini, 2008).

In one elegant study, researchers were able to show how the simple manipulation of a man’s body odour (malodour) altered their self‐confidence as well as their judgements of how visually attractive they were to women (Roberts et al., 2009a). Other researchers, meanwhile, have evaluated both the psychological and physiological effects of perfume on the emotional responses of menopausal women (Abriat et al., 2007). The presentation of a human sex steroid derived compound resulted in increased physiological arousal in women, while decreasing arousal in men (Bensafi et al., 2003). Meanwhile, the fragrance in certain skin creams has also been shown to help relax the wearer, and by so doing may temporarily reduce the facial evidence of wrinkles (Abriat et al., 2004a, 2004b). A number of studies have now shown that both females and males who have been sprayed with either underarm secretions or with one of a number of different synthetic pheromones tend to engage in significantly more everyday sociosexual activities, including sexual intercourse, sleeping next to a partner, formal dating, petting, and affectionate kissing than control participants (e.g., Cowley & Brooksbank, 1991; Cutler, 1987; Cutler et al., 1998; Gower & Ruparelia, 1993; McCoy & Pitino, 2002; see Schaal & Porter, 1991, for a review).

In research conducted in collaboration with scientists working at Unilever Research (makers of the Lynx/Axe deodorant that was mentioned earlier), Roberts et al. (2009a, 2009b) gave one group of young men a control aerosol body spray without odour to use over a period of 72 h. Another group of participants were given a body spray containing a proprietary fragrance oil and an antimicrobial ingredient aimed at reducing malodour (*N* = 35 participants in total). The participants were subsequently instructed to rate how attractive they thought they were to the opposite sex on the basis of photos and short videos that they made specifically to appeal to those of the opposite sex. The results revealed that those given the active body spray felt more self-confident than those with the control spray (presumably due to the reduced malodour). They also rated themselves as being more visually attractive to women. Notice here how it is the effect of odorant on the wearer that is key to the effects that are observed. Although there was no difference between groups in mean attractiveness ratings for the static photos by a panel of females, the same women judged men using the active spray as more attractive in video-clips, suggesting a behavioural difference between the groups. Such results might once again be taken to highlight the methodological concern, highlighted earlier, around whether one chooses to use static or dynamic images of people.

There is also an emerging literature on the possibility of modifying body image by means of olfactory cues in the context of one’s virtual reality (VR) avatar (see Brianza et al., 2019). The latter researchers used a computer-based body visualization tool following one minute of walking in VR, with the scents being sprayed three times during that period. In particular, the results of the latter’s preliminary research suggested that the scent of lemon resulted in the participants reporting that they felt lighter while the vanilla scent made them feel heavier (cf. Hirsch et al., 2003). Once again, here, the ‘meaning’ or association primed by the scent can perhaps best be understood in terms of crossmodal correspondences (see Deroy et al., 2013; Spence, 2011; cf. Risso et al., 2021).

As well as its role in boosting the wearer’s self-confidence, fragrances are sometimes also used to help people get into a particular mood/state of mind. Relevant here actors have, on occasion, anecdotally reported how they sometimes wear a particular fragrance in order to help them get into the role that they are playing (see Spence, 2021b). For instance, according to actress Alla Demidova, the actor Vladimir Vysotsky brought bottles of Parisian perfume to spray on them before performing (as Ranevskaya and Lopakhin, respectively) in *The Cherry Orchard* (Alipaz, 2015).

## Attractiveness as a multisensory construct

Judgments of the attractiveness of a person’s body odour have in some cases been shown to correlate with judgments of the attractiveness (or symmetry) of their face (Rikowski & Grammer, 1999), and even with attractiveness judgments (which are sometimes equated with judgments of mateworthiness) based on the sound of their voice (Cornwell et al., 2004). For instance, Rikowski and Grammer highlighted the existence of a significant correlation between the rated sexiness of a man’s body odour and his facial attractiveness to females. Such findings have led on to discussion in the literature of whether the different senses should be considered as providing redundant, partially redundant, or independent cues to person perception (see Feinberg et al., 2005b; Zuckerman et al., 1991), and specifically to judgments of a person’s beauty (Groyecka et al., 2017). Groyecka et al. discuss these three alternative evolutionary hypotheses aimed at explaining the function of multiple indices of attractiveness. Indeed, Groyecka et al. (2017, p. 1) talk of: ‘the critical need to incorporate cross-modal perception and multisensory integration into future research on human physical attractiveness’. To the extent that it is relevant, the suggestion from those working with other species is that mate selection based on the evaluation of multiple sensory cues (whether or not they happen to be integrated into an overall multisensory assessment) likely represents a more successful mating strategy (Møller & Pomiankowski, 1993). At the same time, however, they also highlight the fact that more research is needed, saying that: ‘The complexity of what people perceive as attractive highlights the need for more research on the multimodal nature of person perception, as challenging as this may be. In addition to studying each modality as if it exists independently of the others (which in the real world it most often does not), researchers have focused disproportionately on visual indicators of attractiveness, underplaying the influence of scent and voice’. (Groyecka et al., 2017, p. 3).

There is also a literature on MHC, a set of genes involved in immune function. This olfactory signature provides clues as to the genetic compatibility (i.e., viability/health) of any potential offspring (Chaix et al., 2008; Havlicek & Roberts, 2009; Havlíček & Roberts, 2013; Penn et al., 2002; Roberts et al., 2005a, 2005b; Santos et al., 2005; Wedekind et al., 1995; Winternitz et al., 2017; see also Jacob et al., 2002). Perhaps unsurprisingly, the growing evidence concerning the informative nature of a person’s body odour in terms of chemosensory communication (Russell, 1976) has also led to growing artistic interest in the idea of the smell dating agency The basic idea here is that people choose their date based on the unfragranced smell of t-shirts after having been worn (Jamieson, 2016). Such an approach has at least some support from the empirical research (Roberts et al., 2011). That said, when Foster (2008) compared the attractiveness ratings made by 44 female participants on sniffing used t-shirts, viewing pictures, or sniffing t-shirts while looking at photos of 21 men, the photos were found to be a much better predictor of overall attractiveness ratings of than were the smelly t-shirts, with the latter only predicting attractiveness amongst the women who were fertile (i.e., those who were not using hormonal birth control). At the same time, however, it has also been shown that women’s preference for dominant male odour may be influenced both by the menstrual cycle and their relationship status (see Havlíček et al., 2005). In a highly cited study, Miller et al. (2007) assessed the tips reported by 18 professional lap-dancers working in gentleman’s clubs over a 60-day period. The results showed that normally cycling participants earned about US$335 per 5-h shift during estrus, US$260 per shift during the luteal phase, and US$185 per shift during menstruation. By contrast, those lap-dancers using contraceptive pills showed no estrous peak in their earnings.

It is not only the role of fertility in attractiveness, though, that separates the sexes. There are also important differences between women and men in terms of scent sometimes having more of an influence over the perception and behaviour of women (Chen & Haviland-Jones, 2000; Doty et al., 1985; though see also Brand & Millot, 2001; Doty & Cameron, 2009; Koelega & Köster, 1974). Women have also been reported to rate smell as a more important sense in mate selection whereas men report valuing visual cues more highly (Havlíček et al., 2008; Herz & Inzlicht, 2002; though see also Johansson & Jones, 2007; Sorokowska, 2013).

## Commercial interest in claims around the effects of fragrance on attractiveness

Given the size of the market, not to mention its longevity, it should not come as any surprise to recognize that there has been a great deal of commercial interest in supporting claims around the effectiveness of personal fragrance (Brady, 1978; Gilbert & Firestein, 2002). Over the years, there has understandably been a great deal of commercial interest by those wishing to capitalize on marketing opportunities associated with functional fragrance claims. Indeed, it is noticeable how many of the studies that have been reported in this review were either funded by the fragrance houses or the home and personal care (HPC) companies, and/or were co-authored by those working within such establishments. At the same time, however, there is also a separate literature relating to patents around the ability of certain olfactory stimuli to influence person perception (e.g., Berliner, 1994; Hirsch, 2006).

For instance, according to a 2006 patent application submitted by Dr. Alan Hirsch, the suggestion is made that the administration of a certain odorant or combinations of odours may provide an effective means of enhancing a woman's self-confidence. The application talks of an odorant or odorant mixture that contains grapefruit, and preferably pink grapefruit, as the dominant odorant. Elsewhere, grapefruit combined with vanilla, and baby powder odorants is suggested to be especially effective*.* The patent makes the claim that smelling such an odorant/odorant mixture for somewhere between three seconds and a minute, but preferably for at least 20 s will reduce a man’s anxiety and also elevate his sense of well-being. In turn, this will result in his having a more positive view of the physical attributes of the woman, for example, perceiving her to be younger than without the fragrance. The magnitude of this crossmodal effect on age perception is suggested to be about 10% (or 4–10 years). Hirsch goes on to suggest that knowing this crossmodal effect of fragrance on male observers may also help to reduce a woman’s anxiety (and at the same time enhance her sense of well-being).

In the research supporting the patent application the influence of impregnating a face mask with grapefruit, grape, or cucumber odour on age judgments are compared. The participants (*N* = 37) were shown photos of 20 individuals and asked to estimate the age of the person shown in each of the images. (The description is, though, unclear about whether this is between- or within-participants design, nor is it clear what proportion of participants were male or female.) The suggestion is that biggest effects observed with men rating women where 6 years reduction mentioned. By contrast, the grapefruit fragrance had no effect of women’s ratings of the age of either male or female photos. However, there is insufficient detail to know to what extent the effects stand up to rigorous statistical analysis.^26^

Hirsch (2006) suggests that a familiar odorant may semantically prime thoughts of people of a given age. In the patent application, Hirsch writes that: ‘The subject's past experience with an aroma and the person in the subject's life who wore the aroma may impact age perception’. He continues: ‘The grapefruit aroma may have acted as a rejuvenator through the mechanism of context-dependent learning’. Giving a concrete example Hirsch then suggests that: ‘a subject whose grandmother often wore lavender may consciously or Sub-consciously associate lavender with octogenarians, whereas the aroma of cotton candy may remind them of more youthful acquaintances. Thus, the aromas, which are traditionally used with older individuals, would act to increase the perceived age where traditionally more youthful smells would serve to induce a rejuvenating effect’. Taken together, the crossmodal effects on age judgments claimed by Hirsch would appear to involve both the indirect effects of mood-induction as well as the semantic priming of age.

In future research it will be interesting to assess the extent to which the laboratory-based crossmodal effects of olfaction on visual ratings of attractiveness, age, or any other attribute, be it of others or of the self, extend to the situations of everyday life (Kirk-Smith & Booth, 1987). In this regard, Roberts et al.’s (2009a) study perhaps comes closest to bridging the sometimes wide gap between well-controlled laboratory studies and ecologically valid real-world studies (see also Sczesny & Stahlberg, 2002).

## Conclusions

According to the majority of the research that has been reviewed here, it would appear that olfactory cues, no matter whether (or even if) they are related to an individual often influence various aspects of person perception, typically operating at a sub-conscious level (see Capparuccini et al., 2010; Li et al., 2007; Novak et al., 2015; see also Coleman, 2021; Holland et al., 2005; Kirk-Smith et al., 1983; Parma et al., 2012). Such crossmodal and multisensory effects can be seen as running counter to the widespread dominance of the visual in everyday life (see Hutmacher, 2019, for a review), and the long history of downplaying the importance of the olfactory sense in humans (see McGann, 2017, for a review).^27^

While acknowledging the importance of the area, it is also true that further research is needed, as highlighted by the following quotes from researchers working in the area. For instance, Roberts et al., (2009a, 2009b, p. 47) note that: ‘Artificial fragrances have been used for thousands of years to manipulate personal odour, but the nature and extent of influences on person perception are relatively unexplored’. (see also Classen et al., 1994). Meanwhile, according to Seubert et al. (2014, p. 6): ‘While olfactory effects on person perception have long been neglected in the laboratory, this study stresses that such effects likely have an important effect on the affective connotation of real-life social interactions and deserve further attention’.

One point that has been stressed repeatedly in reviewing the research is how the participants in the majority of studies have been given no information about, or explanation for, the presence of fragrance in the studies in which they take part. While on the one hand this may help to avoid demand characteristics (namely, participants responding in the way that they expect the experimenter wants them to; see McGlone et al., 2013; Rosenthal, 1964, 1966, 1967) it also makes the demonstration of crossmodal/multisensory effects of olfaction on person perception all the more surprising, suggesting that the effects are automatic and seemingly require no conscious attribution of scent to the individuals that they rate. The fact that sub-threshold smells have been documented to influence person perception (Li et al., 2007) and that crossmodal effects on attractiveness have been shown to influence neural activation in those brain regions that are known to be sensitive to the reward value of facial attractiveness (McGlone et al., 2013) both hint at the existence of a genuinely perceptual crossmodal effect of olfaction on person perception in addition to any olfactorily induced response biases that may sometimes also be picked up.

Perhaps unsurprisingly given what we have seen in this review, those who do not have a functional sense of smell (due to congenital anosmia) have been shown to have anomalous interpersonal relationships (as a result of increased social insecurity; Croy et al., 2013). Given the long-lasting chemosensory impairments reported by many of those who contract Covid-19 (Carfi et al., 2020; Gallagher, 2020) there may be serious, if as yet unacknowledged problems. It turns out that most of us will sniff our hand within a minute of shaking another’s hand (Frumin & Haviv, 2015). Although most of us are unaware of doing it, the suggestion is that this behaviour likely enables us to pick up biologically relevant chemosensory cues about those we meet. At the same time, however, one might be concerned how the elimination of the handshake and the often mandated wearing of facemasks while out in public (Carbon, 2021) is presumably substantially interfering with our normal olfactory perception of others (see Spence, 2020b, for a review). It is in this context that Kirk-Smith and Booth’s (1990) somewhat unusual approach of scenting facemasks with a commercially available fragrance (and banana essence) suddenly appears far more relevant (cf. Hirsch, 2006).

As highlighted by this review, a broad range of crossmodal/multisensory effects of olfactory cues on multisensory person perception (whether that person happens to be the perceived themselves, or someone else) have been documented in the literature. In terms of the putative mechanisms underlying such effects, the emotional response to olfaction (be it mood induction, or crossmodal affective priming) appears to offer one account while others have highlighted the importance of (in)congruency between the sensory stimuli, be it in terms of affective, semantic, or gender-based crossmodal priming. However, it should also be noted that in terms of how people (and other species) go about rating of attractiveness of others, multiple evolutionary accounts have been suggested (Groyecka et al., 2017).

One area that has not been covered in this narrative review concerns the long history of studies that have investigated the evaluative conditioning of affective responses to faces by means of pairing them with odour (e.g., Gottfried et al., 2002; Steinberg et al., 2012; Todrank et al., 1995; see also van Reekum et al., 1999; van den Bosch et al., 2015; and see Syrjänen et al., 2021, for a recent review of olfactory influences on facial attractiveness).

### Implications of crossmodal influences of odour

Those interested in the social aspects of cognitive/experimental psychology (such as the presence versus. absence of another person affecting task performance) might do well to keep in mind the possible role of the body odour/personal scent of the other, no matter whether it happens to be a co-participant, or an experimenter in the testing room, on performance (e.g., Barutchu & Spence, 2020; Lundstrom & Olsson, 2005). One could also imagine how those studies reporting effect of gender of experimenter might also highlight an, as yet, unacknowledged role for olfactory cues.

### Impression management

Gaining a better understanding of the multisensory (especially non-visual) contributions to person perception (e.g., Dalton et al., 2013; Pause et al., 2004) is all the more important when it is realized just how influential visual judgments of personality characteristics such as competency (Todorov et al., 2005), aggressiveness (Carré & McCormick, 2008; Carré et al., 2009), and trustworthiness can be (Stirrat & Perrett, 2010; Wilson & Rule, 2015). Beauty too has been demonstrated to bias outcomes in the job market, not to mention life more generally (e.g., Hamermesh & Biddle, 1994; Maestripieri et al., 2016; Mulford et al., 1998), in part via the halo effect mentioned earlier. And while there is an analogous literature on the consequences of vocal qualities, such as pitch (e.g., in terms of the electability of politicians; Klofstad, 2016; Klofstad & Anderson, 2018; Tigue et al., 2012) and fundamental and formant frequency (e.g., on ratings of attractiveness; Feinberg et al., 2005a; cf. Leongômez et al., 2017), there has been virtually no research looking at crossmodal judgments of voice being influenced by olfactory cues (though see Aglioti & Pazzaglia, 2011; Ferdenzi et al., 2016; Groyecka et al., 2017; Wesson & Wilson, 2010, 2011, for the slowly emerging interest in crossmodal interactions between this particular pair of senses). Nevertheless, the many crossmodal effects of body odour and fragrance on multisensory person perception undoubtedly support earlier claims regarding the importance of scent to multisensory impression management (e.g., Baron, 1981, 1983; Dabbs et al., 2001; Fiore, 1992; Higuchi et al., 2005; Kirk-Smith & Booth, 1987; König, 1972; Nezlek & Shean, 1995; cf. Lobmaier et al., 2020), while at the same time contradicting earlier claims that olfaction was irrelevant to social interaction (see Argyle, 1975, pp. 227, 327).

### Digital olfaction and the mediated self

Looking to the future, there are many in the world of human–computer interaction who are actively considering the potential inclusion of scent in digital devices (e.g., Bodnar et al., 2004; Braun et al., 2016; Brewster et al., 2006; Dobbelstein et al., 2017), given the explosion in online dating, not to mention the release of scent-enabled mobile phones (see Gray, 2007). It is, though, important to highlight the very limited range of scents that even the latest olfactorily enabled consumer devices can achieve (see Spence et al., 2017, for a critical review). As such, the most likely usage scenario might be to fill a scent-dispensing plug-in with your loved one’s perfume/aftershave and have a squirt be dispensed whenever they call you on their mobile device. However, while such a scent is likely to be positively valenced for the perceiver, despite almost four decades of research on scent’s effect on person perception, it is still not known whether it will convey exactly the same benefits in terms of reducing anxiety, etc., that the smell of a loved one’s body odour has been shown to do (Granqvist et al., 2018; Hofer et al., 2018; McBurney et al., 2006; cf. O’Brien, 2018). This, then, is just one of the questions still awaiting resolution in this most intriguing of research areas at the borders of basic and applied cognitive/perceptual research in experimental psychology.

## Data Availability

Not applicable.

## Footnotes

1 As Stoddart (1990) put it: ‘human beings behave as if they are afraid of smelling like human beings, for human beings smell bad’.

2 To give some sense of the size of the global market for fragranced facial and body products, according to Small and Green (2012), it was estimated at an impressive US$ 425.8 billion in 2011 alone (see Aikman, 1951, for a historical perspective; and http://www.leffingwell.com/top_10.htm for more recent figures).

3 Though, sadly, the Venus de Milo and Mona Lisa were not amongst them. And, what is more, the bespoke scents themselves were only available for sale in the gift shop, and not introduced to the relevant galleries.

4 In one study, for example, those exposed to a disgusting negative ambient odour (a commercially available ‘fart spray’ comprising hydrogen sulphide and ammonia) gave harsher judgments regarding several types of moral vignettes (for example, sex between first cousins) than those exposed to the control scent (Schnall et al., 2008).

5 Nor is it altogether clear what the participants in the studies considered the relationship between fragrance and visual stimuli to be, or even whether they gave any thought to the matter.

6 At the same time, however, and as we will see later, odours do not always have to be perceived consciously in order to influence human performance (e.g., Holland et al., 2005; Li et al., 2007).

7 A perfume that Paukner (1965) once memorably described by as being similar to an ‘ideal erogenous perfume’.

8 Relevant here, both odorants were judged as being equally pleasant, hence ruling out a valence-based explanation for the different pattern of results obtained with the two scents.

9 This world-famous brand aimed at young men goes by the brand name of *Axe* in many countries.

10 The *Lynx* fragrance and the synthetic body odour scents were both assumed to be more body-relevant to the faces of the young men who were being evaluated that either the smell of geranium and rubber. However, it could be argued that in order for the female participants to code the *Lynx* fragrance as body-relevant, they would first have needed to recognize the fragrance, presented without any other identifying information, as the smell of a famous young man’s deodorant brand.

11 The halo effect refers the suggestion that attractive individuals are also considered to have a variety of other positive personal attributes (see also Carragher et al., 2021).

12 It is worth noting that the caramel odour typically takes on innately pleasurable associations with the sweet-taste with which it is normally paired, whereas the aroma of liquorice is more likely to be associated with salty/bitter tastes instead (Stevenson & Boakes, 2004).

13 Note that the liquorice and caramel conditions are mislabelled in Risso et al. (2021, Figure 4), as confirmed by pers. comm. with first author dated May 9th, 2021.

14 One might even consider what kind of semantic links may be established in the mind of the consumer with a fragrance that happens to be heavily endorsed by a celebrity (Mahdavi et al., 2019).

15 Four male and four female faces, each displaying neutral, happy, and disgusted facial expressions. Using these pictures, 16 unique video clips were created lasting 3 s each. In each video clip, a neutral facial expression developed gradually into either a happy or a disgusted expression. The transformation of faces was therefore not exactly like looking at a natural dynamic face.

16 Intriguingly, while there were visual-olfactory stimulus combinations that were either congruent (negative-negative) or incongruent (negative-neutral) in terms of their emotion, few, if any, of the combinations could be considered to be semantically congruent, excepting, perhaps, the synthetic BO, fearful face combination.

17 This consistent with the fact that: ‘perfumes are generally marketed as having the ability to enhance sexual attractiveness’ (Berliner et al., 1991, p. 671).

18 Along somewhat similar lines, some commentators have been tempted to suggest that hedonically valenced odours need not modulate facial attractiveness, but might rather affect other affective components of interpersonal perception , for example, perceived sympathy (König, 1972).

19 Note that for various reasons, olfaction (one of the chemical senses) and vision (what Novak et al., 2015, call a ‘physical’ sense, and Spence, in press, has described as a spatial sense) may potentially show less interaction/confusion.

20 Speed-dating events might provide an ideal real-world situation to collect some of the relevant data.

21 Apparently aware of how easily scents could be misattributed, the young men of the French court of centuries gone by would fragrance their apartments in order to try and woo the ladies (Corbin, 1986, p. 74). Corbin (1986, p. 77) also mentions those who would wear fragranced clothing, with the presumption once again seemingly being that whoever became aware of that fragrance would attribute it to the person rather than their garb. Relevant here, a marketing study by Spangenberg et al. (2006) highlighted the impact of gender-congruent ambient scent on the on customers’ approach-avoidance behaviours in the context of a clothing retail store. Sometimes, in other words, the gendered scent may be associated with the location, or clothing, rather than necessarily with a particular individual.

22 In the project Actual Odor, the artist Angela Ellsworth wore a jersey cocktail dress soaked in her own urine for the duration of the opening reception for the Token City installation (a subway simulation) by artist Muriel Magenta at the Arizona State University Art Museum (1997; Drobnick, 2006).

23 Thus contrasting with Graham and Jouhar’s (1980, p. 97) early claim that: ‘All in all, it would appear that sense of smell is fairly unimportant in that we do not rely very much on body odours in their natural form nor is it socially acceptable to emit such body odours. However, the reverse is the case for manufactured odours in the form of perfumes, deodorants or odours indicating cleanliness.’.

24 Rosebury (1969, p. 208) poses an interesting question: "Maybe we ought to stop at times to wonder why we like flowers or coconuts or little Asiatic deer or the guts of a sperm whale; couldn't we learn to love the smell of healthy sweat of men and women?" Or as Desmond Morris (1967), puts it in "The Naked Ape": "The female who so assiduously washes off her own biological scent, then proceeds to replace it with commercial 'sexy' perfumes which in reality are no more than diluted forms of the products of the scent glands of other, totally unrelated mammalian species.”

25 And one should not forget how ‘Napoleon, in one of his more infamous letters to Josephine, begged her not to bathe, for he wanted to enjoy her body odor to the fullest’. (Tanabe, 2004).

26 It should also be noted that some of Dr. Hirsch’s claims regarding the power of olfaction have not gone uncontested in the courts (see Deardorff & King, 2014).

27 For instance, almost a century ago, Herrick (1924) classified primates in general and human in particular as ‘microsmatic’, on the basis that olfaction played a minor role in their behaviour (though see Schaal & Porter, 1991, for the contrary position).
